# First genome report and analysis of chicken H7N9 influenza viruses with poly-basic amino acids insertion in the hemagglutinin cleavage site

**DOI:** 10.1038/s41598-017-10605-6

**Published:** 2017-08-30

**Authors:** Jidang Chen, Jipei Zhang, Wanjun Zhu, Yishan Zhang, Hualong Tan, Minfang Liu, Mingsheng Cai, Jiaren Shen, Hinh Ly, Jianhong Chen

**Affiliations:** 1 School of Life Science and Engineering, Foshan University, Foshan, China; 2 Department of Veterinary & Biomedical Sciences, College of Veterinary Medicine, University of Minnesota, Twin Cities, Minnesota USA; 3 Department of Pathogenic Biology and Immunology, Sino-French Hoffmann Institute, School of Basic Medical Science, Guangzhou Medical University, Guangzhou, Guangdong, China; 4 Shanghai Municipal Center for Disease Control and Prevention (SCDC), Shanghai, China

## Abstract

We report the full-length sequence of two chicken source influenza A (H7N9) viruses found in Guangdong live poultry market (LPM) during the most recent wave of human infections (from October 2016 to the present time). These viruses carry insertion of poly-basic amino acids (KGKRTAR/G) at the protease cleavage site of the HA protein, which were previously found in the highly pathogenic (HP) human influenza A (H7N9) [IAV(H7N9)] strains. Phylogenetic analysis of these two novel avian influenza viruses (AIVs) suggested that their genomes reassorted between the Yangtze River Delta (YRD) and Pearl River Delta (PRD) clades. Molecular clock analysis indicated that they emerged several months before the HP human strains. Collectively, our results suggest that IAV(H7N9) viruses evolve in chickens through antigenic drift to include a signature HP sequence in the HA gene, which highlights challenges in risk assessment and public health management of IAV(H7N9) infections at the human-animal interface.

## Introduction

From March 2013 to March 2017, annual epidemics of IAV(H7N9) in China resulted in 1,329 human infections as reported to the World Health Organization (WHO) by the National Health and Family Planning Commission of China and other regional sources^1, 2^. The first wave of infections in spring 2013 (W1, weeks 7/2013–40/2013) included 135 cases; whereas 319 cases were reported during the second wave (W2, weeks 41/2013–40/2014), 223 cases during the third (W3, weeks 41/2014–40/2015), and 121 cases in the fourth wave (W4, weeks 41/2015–40/2016). A fifth wave of infections started in October 2016 (W5, week 41/2016), with 235 cases reported as of 24 January 2017^3^. During the first four epidemics, 88% of patients developed pneumonia, 68% were admitted to an intensive care unit, and 41% died^4^. During the ongoing fifth wave, 460 human IAV(H7N9) infections have been reported, including 453 in mainland China; six associated with travel to mainland China from Hong Kong (four cases), Macao (one) and Taiwan (one); and one in an asymptomatic poultry worker in Macao^1^. Previous analyses indicated that IAV(H7N9) viruses have diverged into two distinct genetic lineages, including the YRD and PRD clades^5^.

All IAV(H7N9) viruses identified from the first four epidemics harbored a low pathogenic signature, bearing the KGR/G sequence, in the host protease cleavage site on the viral HA protein^6, 7^. However, human samples recently reported from the fifth epidemic contain the KRKRTAR/G or KGKRIAR/G motif with insertion of multiple basic amino acids in the HA cleavage site that is characteristic of HP avian influenza (HPAI) viruses^5, 8, 9^. However, to date (April 2017), there is no report of similar HPAI HA cleavage site found in avian IAV(H7N9) viruses.

According to the WHO, from Jan-19 to Feb-7, 2017, 304 human infected cases have been reported, among them 47.4% (144/304) had a history of avian contact^1^. From Feb-24 to Mar-7, there were 57 cases, with 75.4% (43/57) known to have avian contact^2^. Therefore, avian contacts are considered to be closely related to human IAV(H7N9) cases. Previous findings have also hinted that live poultry markets (LPMs) play a key role in multiple subtype AIV infecting humans^10, 11^. We therefore have recently conducted a survey of AIV infections among chickens, ducks, geese and pigeons sold in a LPM in Guangdong province and have found that 2 samples from chickens were positive for AIV. Importantly, our phylogenetic analysis has shown that these novel avian viruses are reassortants of the two distinct genetic lineages of the YRD and PRD clades and that their HA proteins contain the KGKRTAR/G motif with insertion of poly-basic amino acids at the HA cleavage site, which has previously been shown to be among the sequences of HP human influenza virus strains. We therefore propose that this HA sequence change observed in the novel AIVs in our study might serve as a potential precursor to those found in HP human H7N9 viruses.

## Results

### Sample information

On February 25^th^, 2017, during the fifth IAV(H7N9) epidemic, from a LPM of Foshan City in the Guangdong province of China, we collected a total of 200 tracheal swab samples from 50 chickens, 50 ducks, 50 geese and 50 pigeons. These birds showed no significant clinical signs. After real time RT-PCR, 2 samples were found to be H7 HA gene positive. These two swab samples were collected from chickens kept in separate cages and sold by different vendors that showed no significant clinical signs. The two strains were named A/Chicken/Guangdong/J1/2017(H7N9) and A/Chicken/Guangdong/J2/2017(H7N9) (abbreviated as CK/J1 and CK/J2, respectively). Accession numbers KY855515-KY855530 of these two strains were obtained after whole genome sequences were submitted to NCBI GenBank.

### Viral Proteins and Molecular Characteristics of the Two Novel AIVs

The molecular signatures of CK/J1 and CK/J2 associated with host adaptation, receptor specificity, and potential pathogenesis and antiviral resistance were assessed, and compared with three recently described HP human IAV(H7N9) isolates, including A/Guangdong/17SF006/2017(H7N9), A/Qingyuan/GIRD1/2017(H7N9) and A/Taiwan/1/2017(H7N9), abbreviated as GD/17SF006, QY/GIRD1 and TW/1, respectively, with amino acid insertion in the HA cleavage site (Table 1). Q226L/I and G228S substitutions in the HA protein, which are the two main mutations contributing to the high-affinity binding of viruses to human receptors, were not identified in the two novel AIVs. However, several substitutions that may increase the binding ability of the viruses to the human α-2,6-linked sialic acid receptor in HA were detected, namely S138A, T160A, and G186V. NS1 E172K, which induces viral replication in mammalian cells, was found in both chicken and human influenza viruses^12^. Virulence-related signatures P42S and D92E substitutions in the NS1 protein, were also identified. M2 S31N, which confers resistance to amantadine and rimantadine, was found in all these viruses. However, mutations of the NA protein that may cause Oseltamivir resistance, such as E119V and I222L, were not detected in these viruses. Different from human strains, the two AIVs found in this study still harbored known avian influenza virus signatures, e.g. the PB2 E627K mutation was found in 3 human strains, but not in CK/J1 and CK/J2. Meanwhile, PA K356R did not occur in CK/J1 but harbored by the CK/J2 and 3 human viruses (Table 1).

**Table 1:** Molecular analysis of A/Chicken/Guangdong/J1/2017(H7N9) and A/Chicken/Guangdong/J2/2017(H7N9).

Protein	Position	Mutation	Avian Strains		Human Strains			Biological feature altered by mutations
			CK/J1	CK/J2	GD/17SF006	QY/GIRD1	TW/1	
**NS1**	42	P-S	S	S	S	S	S	P42S increases virulence in mice^29^
	92	D-E	D	D	D	D	D	Altered virulence in mice^30^
	205	N-S	S	S	S	S	S	Altered antiviral response in host^31^
	210	G-R	G	G	G	G	G	Altered antiviral response in host^13^
**M2**	31	S-N	N	N	N	N	N	S31N is known to confer resistance to amantadine and rimantadine^32^
**NA**	119	E-V	E	E	E	E	E	Oseltamivir resistance (N2 numbering)^33^
	222	I-L	I	I	I	I	I	Oseltamivir resistance (N2 numbering)^33^
	292	R-K	R	R	K	K	K	R292K is known to confer resistance to oseltamivir and zanamivir (N2 numbering)^34^
**HA**	CS	Basic aa insertion	PEVPK**G**KRTARGL	PEVPK**G**KRTARGL	PEVPKRKRTARGL	PEVPKRKRTARGL	PEVPKRKRTARGL	
	138	S-A	A	A	A	A	A	S138A increases virus binding to α-2,6-linked sialic acid receptor (human receptor) (H3 numbering)^20^
	160	T-A	A	A	A	A	A	T160A increases binding to α-2,6-linked sialic acid receptor (human receptor) (H3 numbering)^21^
	186	G-V	V	V	V	V	V	G186V increases binding to α-2,6-linked sialic acid receptor (human receptor) (H3 numbering)^22^
	226	Q-L	Q	Q	Q	Q	Q	Q226L increases binding to α-2,6-linked sialic acid receptor (H3 numbering)^25^
	228	G-S	G	G	G	G	G	Increased binding to human-type influenza receptor (H3 numbering)^35^
**PA**	100	V-A	A	A	**V**	**V**	A	Species-associated signature positions^36^
	336	L-M	L	L	L	L	L	Increased polymerase activity in mice^37^
	356	K-R	**K**	R	R	R	R	Species-associated signature positions^38^
	409	S-N	N	N	N	N	N	Species-associated signature positions^36^
**PB1**	368	I-V	V	V	V	V	V	Increased transmission in ferrets^18^
**PB1-F2**	66	N-S	N	N	N	N	N	Induction of apoptosis^39^
**PB2**	627	E-K	**E**	**E**	K	K	K	
	701	D-N	D	D	D	D	D	D701N increases adaptation to mammals^18^
	702	K-R	K	K	K	K	K	Species-associated signature positions^40^

Abbreviation: CK/J1, CK/J2, GD/17SF006, QY/GIRD1, TW/1 represent for IAV(H7N9) strains A/Chicken/Guangdong/J1/2017(H7N9), A/Chicken/Guangdong/J2/2017(H7N9), A/Guangdong/17SF006/2017(H7N9), A/Guangdong/GIRD1/2017(H7N9) and A/Taiwan/01/2017(H7N9) respectively. aa short for amino acids; HA short for hemagglutinin; H3 short for hemagglutinin subtype 3; M2 short for matrix 2 protein; NA short for neuraminidase; N2 short for neuraminidase subtype 2; NS short for non-structural protein; PA short for polymerase acidic protein; PB short for polymerase basic protein; PB1-F2 short for polymerase basic protein 1 alternate reading frame 2. Underlined characters indicating the different amino acids among viruses.

### Additional Amino Acid Insertion in HA Cleavage Sites of the Two Novel AIVs

**Figure 1:**
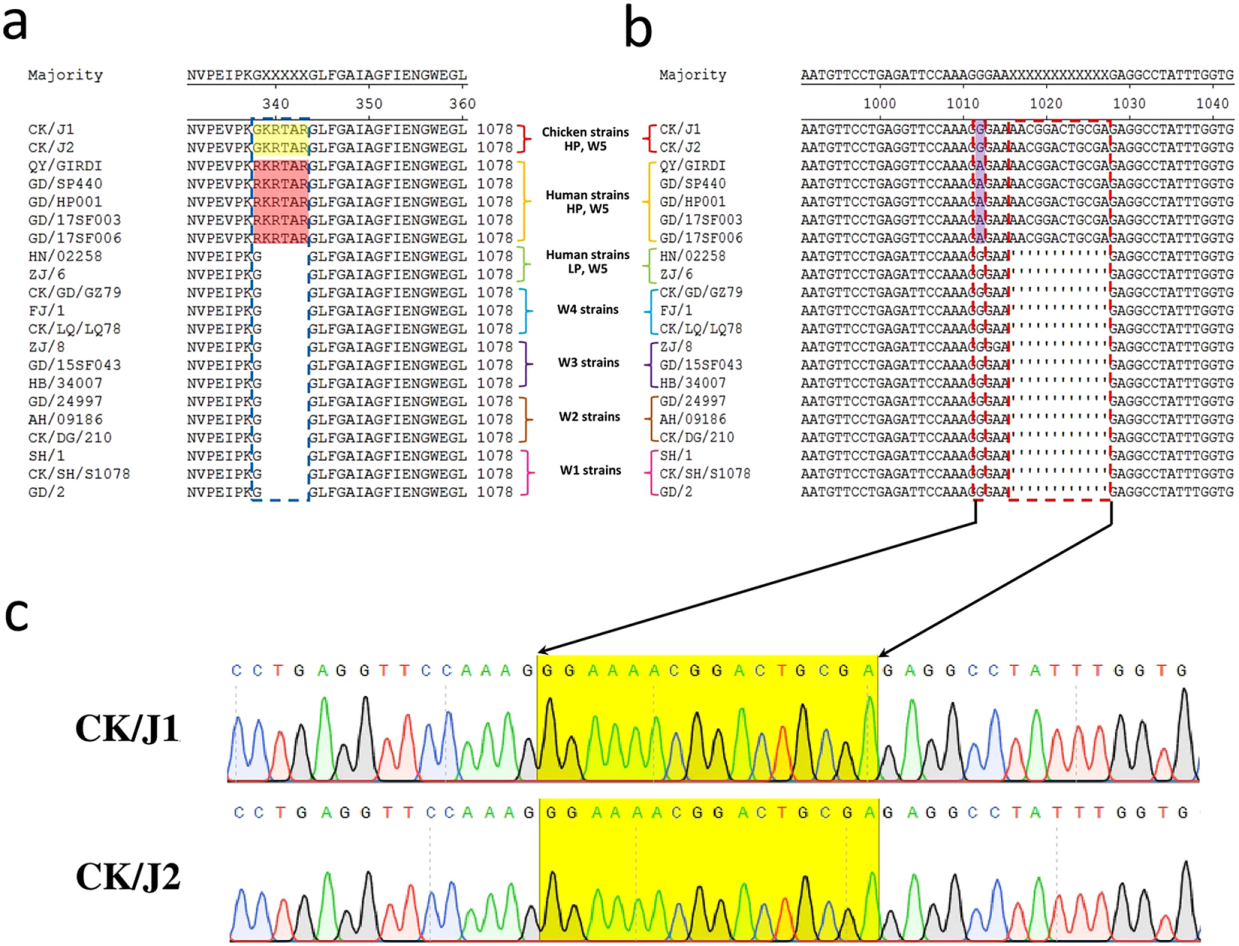
Molecular analysis of the cleavage site of HA amino acid sequences (Fig. 1a) and nucleotide sequences (Fig. 1b) of the CK/J1 and CK/J2 viruses, and respective H7N9 virus strains found in first to fifth epidemics (W1~W5). Numbering starts at the N-terminus/open reading frame (ORF, 1~1695nt) of HA. Only the amino acid and nucleotide sequences near the cleavage site regions are shown. In Fig. 1a, the amino acid insertion region is shown by blue break frame; the inserted amino acids of CK/J1 and CK/J2 are shown in yellow shadow; the high pathogenic human strains are shown in red shadow. Figure 1b, nucleotide mutation and insertion are shown in red break frame. Figure 1c, cleavage site sequencing peak maps of CK/J1 and CK/J2. Yellow shadow showing the mutation and nucleotide insertion in the cleavage site. Abbreviations: CK/J1(A/Chicken/Guangdong/J1/2017), CK/J2(A/Chicken/Guangdong/J1/2017), QY/GIRD1(A/Qingyuan/GIRD1/2017), GD/SP440(A/Guangdong/SP440/2017), GD/HP001(A/Guangdong/HP001/2017), GD/17SF003(A/Guangdong/17SF003/2016), GD/17SF006(A/Guangdong/17SF006/2017), HN/02258(A/Hunan/02258/2017), ZJ/6 (A/Zhejiang/6/2017), CK/GD/GZ79(A/Chicken/Guangdong/GZ79/2016), FJ/1(A/Fujian/1/2016), CK/LQ/LQ78(A/Chicken/Longquan/LQ78/2016), ZJ/8(A/Zhejiang/8/2015), GD/15SF043(A/Guangdong/15SF043/2015), HB/344007(A/Hebei/344007/2015), GD/24997(A/Guangdong/24997/2013), AH/09186(A/Anhui/09186/2014), CK/DG/210(A/Chicken/Dongguan/210/2014), SH/1(A/Shanghai/1/2013), CK/SH/S1078(A/Chicken/Shanghai/S1078/2013), GD/2(A/Guangdong/2/2013), LP (Low Pathogenic), HP (High Pathogenic). Amino acid and nucleotide sequence alignments were performed with MegAlign 7.0 (DNASTAR Inc. USA).

In the first four epidemic waves (from Mar 2013 to Oct 2016), all reported viruses had the low pathogenic cleavage site in the amino acid sequence **PEIPKG/GLF** of the HA proteins (Fig. 1a). However, during the fifth wave, 5 human IAV(H7N9) strains isolated in Guangdong, A/Qingyuan/GIRD1/2017, A/Guangdong/SP440/2017, A/Guangdong/HP001/017, A/Guangdong/17SF003/2017 and A/Guangdong/17SF006/2017 (or QY/GIRD1, GD/SP440, GD/HP001, GD/17SF003 and GD/17SF006, respectively) had an insertion of 3 basic amino acid residues (RKR) in the cleavage site connecting the HA1 and HA2 peptide regions, carrying the **PEVPK** **RKR** **TAR/GLF** sequence, which is a signature of HPAI viruses^8^. The two novel AIVs CK/J1 and CK/J2 also had similar insertions in the cleavage site of HA protein, but they slightly differed from the human strain signature. The chicken viruses carried the **PEVPK** **GKR** **TAR/GLF** sequence with only 2 basic amino acid residues inserted (KR) (Fig. 1a). Such amino acid mutations corresponded to nucleotide changes. Specifically, 12 additional nucleotides (AACGGACTGCGA) were found in CK/J1, CK/J2 and the 5 new human isolates, but not in the first 4 epidemics (W1 to W4) strains (Fig. 1b). A nucleic acid mutation G1012A (H7 numbering) was also found in the human strains, but not in CK/J1, CK/J2 and other H7 viruses with the LP cleavage site (Fig. 1b). The HA sequence peak map on the cleavage site regions of CK/J1 and CK/J2 showed a single peak for each nucleotide, which indicates that sequencing results are trustworthy (Fig. 1c). This was the first demonstration of such a molecular sequence-signature characteristic in chicken IAV(H7N9) viruses since their emergence in 2013, according to the alignment of viral HA sequences available in the GISAID database.

### Genetic Reassortment Occurred in Both Novel Human and Avian IAV(H7N9) Strains

**Figure 2:**
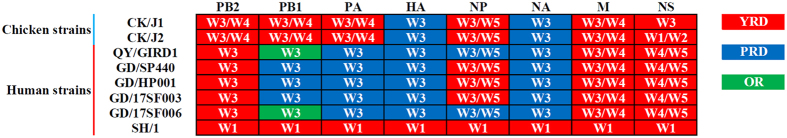
Genotypes of eight gene segments of H7N9 influenza A viruses from Feb 2013 to Jan 2017. Figure 2 shows genotype models of chicken and human strains with HP characteristics. Red squares indicate genes belonging to the Yangtze River Delta clade, blue squares represent genes of the Pearl River Delta clade and green squares represent genes belonging to clade from other regions. Abbreviations: YRD (Yangtze River Delta), PRD (Pearl River Delta), OR (other regions); W1, W2, W3, W4 and W5 (1^st^ to 5^th^ wave H7N9 epidemics, respectively). PB2 (polymerase basic protein 2 gene), PB1 (polymerase basic protein 1 gene), PA (polymerase acidic protein gene), HA (hemagglutinin gene), NP (nucleoprotein gene), NA (neuraminidase gene), M (matrix protein gene), and NS (non-structural protein). CK/J1(A/Chicken/Guangdong/J1/2017), CK/J2(A/Chicken/Guangdong/J2/2017), QY/GIRD1(A/Qingyuan/GIRD1/2017), GD/SP440(A/Guangdong/SP440/2017), GD/HP001(A/Guangdong/HP001/2017), GD/17SF003(A/Guangdong/17SF003/2016), GD/17SF006(A/Guangdong/17SF006/ 2017) and SH/1(A/Shanghai/1/2013).

Analysis was performed based on sequences of each CK/J1 and CK/J2 segment, and those retrieved from the database of GISAID. The sequences of representative IAV(H7N9) viruses sampled during epidemic waves 1 to 5 (Feb 2013 to Jan 2017) from different geographical regions of China originated from avian and human specimens (Supplementary Table S1). Phylogenetic trees were constructed for each gene segment (PB2, PB1, PA, HA, NP, NA, M, and NS) of IAV(H7N9) viruses (Supplementary Figures S1–S4). On the phylogenetic trees, these viruses formed two main distinct genetic clades, including YRD and PRD. As indicated by the trees, some viruses originated from other regions (OR), such as Beijing, Hebei, Jilin, Fujian, Hunan, Jiangxi and Xinjiang *et al*., formed small clades among the YRD and PRD lineages (Supplementary Figures S1–S4). Two glycoprotein genes, HA and NA, of the novel AIVs (CK/J1 and CK/J2) and human strains recently found in Guangdong province, all belong to the PRD lineage. As for genetic relationship, these viruses closely related to W3 strains (Fig. 2, Supplementary Figures S2 and S3). The polymerase tripartite genes (PB2, PB1 and PA) of CK/J1 and CK/J2 were derived from the YRD clade, and close to W3 and W4 viruses. Similar with the two chicken viruses, PB2 of the five Guangdong human isolates also originated from the YRD clade in W3 to W4. However, PA genes of the 5 human strains and PB1 of GD/SP440, GD/HP001 and GD/17SF003 were located in the PRD W3-like lineage, while QY/GIRD1 and GD/17SF006 were located in the OR clade W3-like branch (Fig. 2, Supplementary Figures S1 and S2). NP genes of the two avian and five human strains were closely related to W3 to W5 strains. Except QY/GIRD1 and GD/17SF006 strains, which derived from PRD, the NP genes of other 5 strains originated from the YRD clade (Fig. 2, Supplementary Figure S3). The M and NS genes of all 7 viruses derived from the YRD clade. The M genes of avian and human strains had close genetic relationship with W3 to W4 viruses (Fig. 2, Supplementary Figure S4). Although all NS genes originated from the YRD clade, they derived from different epidemic periods. CK/J1 was genetically closely related to the W3 viruses, and CK/J2 to the W1 to W2 strains. NS genes of the 5 human viruses were from W4 strains. As a common ancestor of IAV(H7N9) virus, all segment genes of A/Shanghai/1/2013(H7N9) (termed as SH/1) derived from the YRD clade. From the phylogenetic trees, polymerase tripartite and NS genes were close to avian strains instead of human ones (Fig. 2, Supplementary Figures S1, S2 and S4).

### Genetic Diversity of IAV(H7N9)’s HA and NA genes in China between March 2013 and January 2017

**Figure 3:**
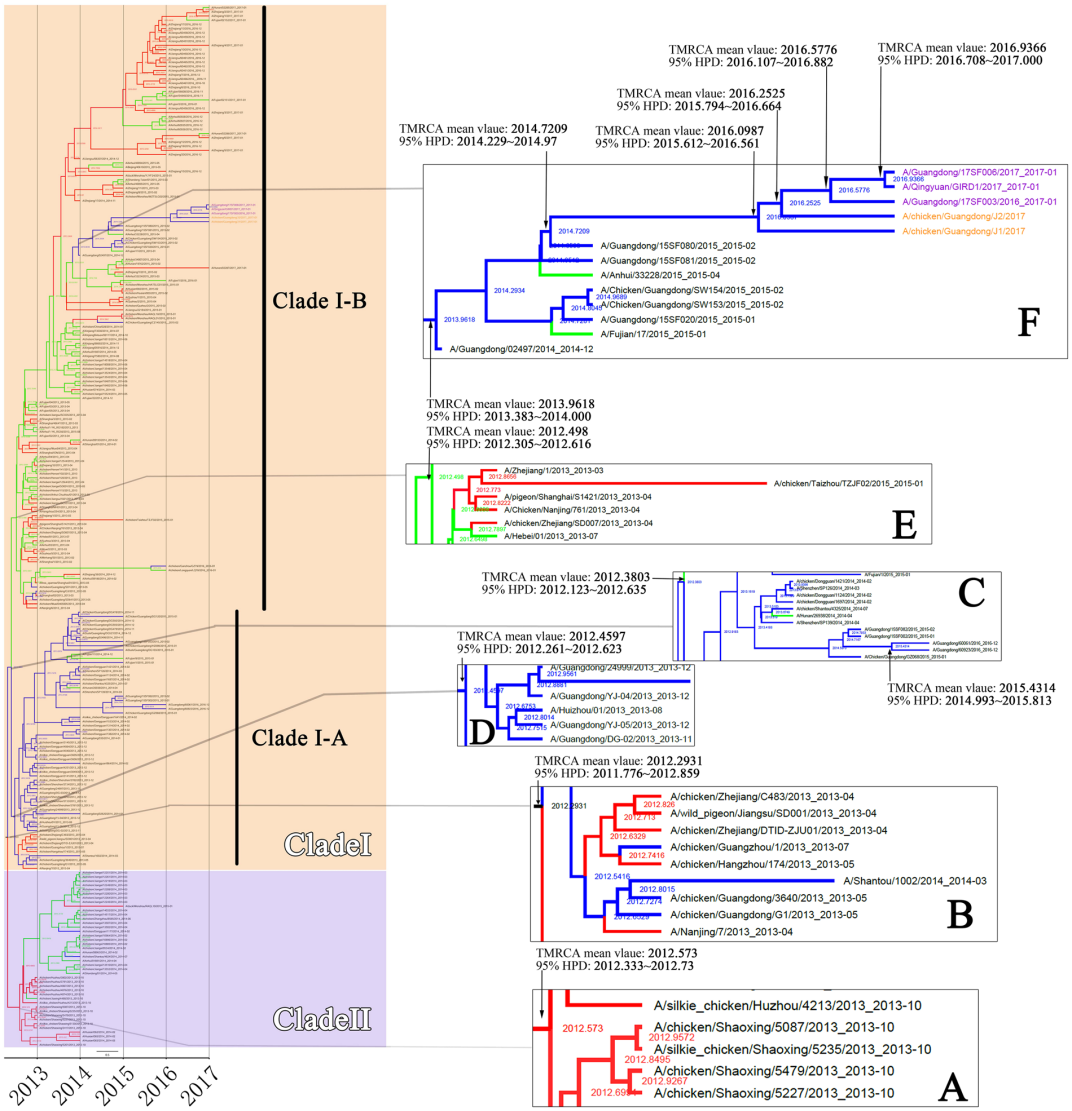
Bayesian maximum clade credibility (MCC) phylogeny of H7 gene sequences of waves 1 to 5. Viruses isolated in the Yangtze River Delta, Pearl River Delta, and other regions are highlighted in red, blue, and green, respectively. BEAST tree of H7N9 viruses estimated using HA gene sequences. Orange shadow indicates Clade I of H7N9 viruses; purple represents Clade II. Figure 3A, time of most recent ancestor (TMRCA) of Clade II; Fig. 3B, TMRCA of the entire tree. Figure 3C, TMRCA of Clade I. Figure 3D, TMRCA of the Clade I-A lineage. Figure 3E, TMRCA of the Clade I-B lineage. Figure 3F, TMRCA of the lineage containing H7N9 viruses detected in avian or humans with HP characteristic cleavage site. Purple characters, human HP strains; orange characters, chicken HP strains detected in this study.

**Figure 4:**
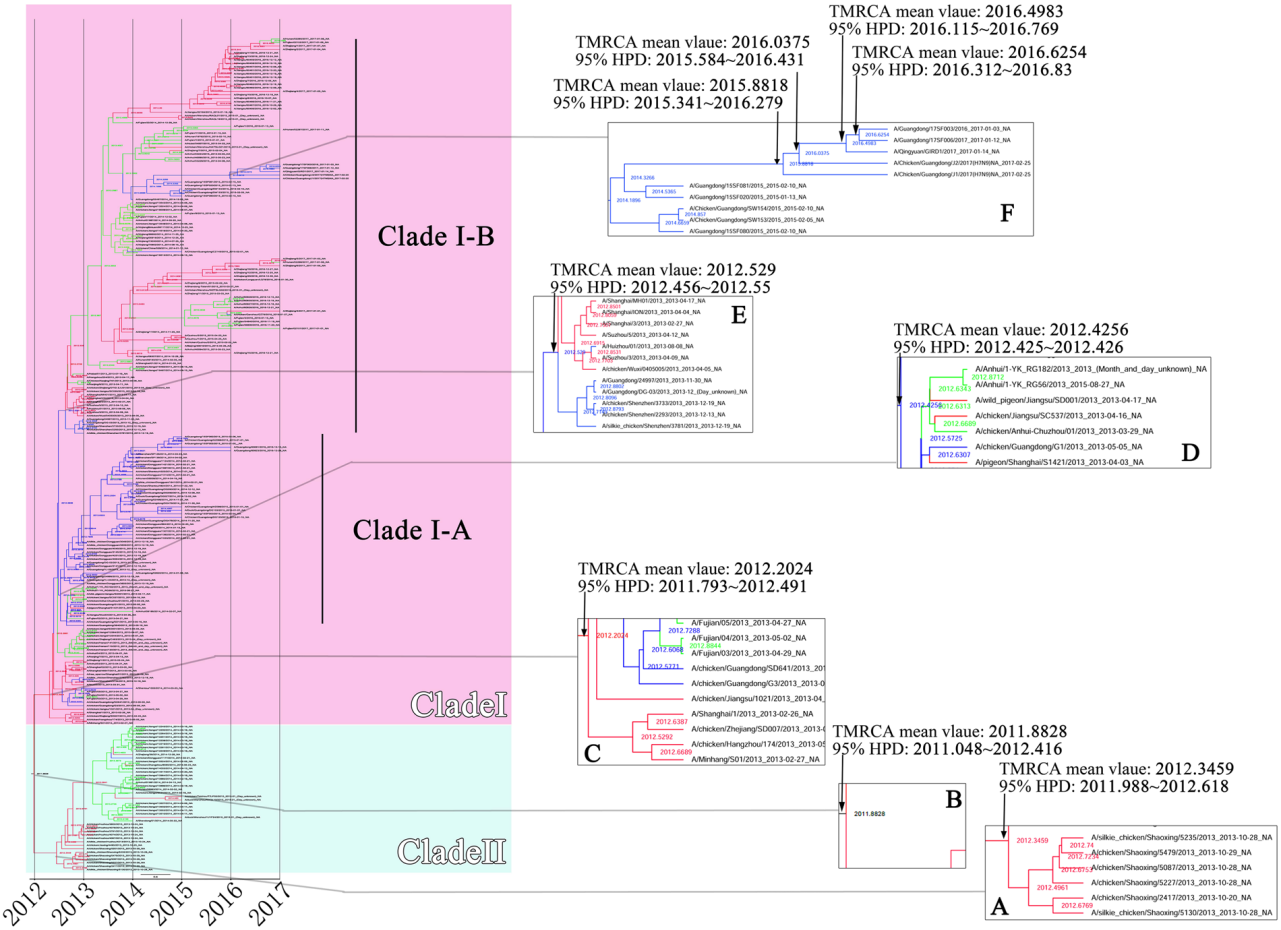
Bayesian maximum clade credibility (MCC) phylogeny of N9 gene sequences of waves 1 to 5. Viruses isolated in the Yangtze River Delta, Pearl River Delta, and other regions are highlighted in red, blue, and green, respectively. BEAST tree of H7N9 viruses estimated using NA gene sequences. Pink shadow indicates Clade I of H7N9 viruses; light blue represents Clade II. Figure 4A, time of most recent ancestor (TMRCA) of Clade II; Fig. 4B, TMRCA of the entire tree. Figure 4C, TMRCA of Clade I. Figure 4D, TMRCA of the Clade I-A lineage. Figure 4E, TMRCA of the Clade I-B lineage. Figure 4F, TMRCA of the lineage containing H7N9 viruses detected in avian or humans with HP characteristic cleavage site.

Analysis of the time of the most recent common ancestor (TMRCA) of HA and NA genes of IAV(H7N9) suggests that TMRCA mean values of HA and NA genes were 2012.2931 and 2011.8828, respectively, with 95% highest posterior density (HPD) interval of HA and NA genes were 2011.776~2012.859 and 2011.048~2012.416, respectively (Figs 3B and 4B). From W1 to W5, both HA and NA genes were clustered into two big clades, namely Clades I and II. The HA and NA genes which clustered into Clade II originated from the YRD and OR regions. The TMRCA of two glycoproteins genes in Clade II was estimated from March 2012 to July 2012 with the mean TMRCA values of HA and NA genes were 2012.573 and 2012.346, respectively, and 95% HPD of HA and NA genes were 2012.333~2012.737 and 2011.988~2012.618, respectively (Figs 3A and 4A). Most viruses clustered in this clade were of the avian source strains. Two genes which clustered in Clade I were in all affected regions (YRD, PRD and OR). TMRCA of both two genes in Clade I was estimated to be Jan 2012 to May 2012 with the mean TMRCA values of HA and NA genes were 2012.3803 and 2012.2024, respectively, and 95% HPD of HA and NA were 2012.123~2012.635 and 2011.793~2012.491, respectively (Figs 3C and 4C). In Clade I, IAV(H7N9)’s HA genes developed into two lineages, i.e. Clade I-A and Clade I-B. Similar with the HA genes, after April 2012, most of the NA genes of IAV(H7N9) in Clade I formed two main lineages (also termed as Clade I-A and Clade I-B). Both HA and NA genes of most viruses included in Clade I-A were from the PRD region, with most of avian source. This lineage was estimated to start around May 2012 (mean TMRCA values of HA and NA genes were, 2012.4597 and 2012.4256, respectively; 95% HPD of HA and NA were 2012.261~2012.623 and 2012.425~2012.426, respectively) (Figs 3D and 4D). The Clade I-B was estimated to start around June 2012 (mean TMRCA values of HA and NA genes were 2012.498 and 2012.529, respectively; 95% HPD of HA and NA were 2012.305~2012.616 and 2012.456~2012.550, respectively) (Figs 3E and 4E). In Clade I-B, most viruses were from YRD and OR.

**Figure 5:**
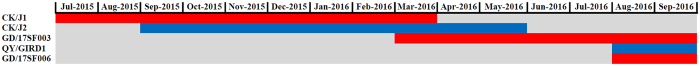
Map of estimated divergence time of HA gene of the two chicken HP H7N9 strains (CK/J1 and CK/J2) and three human HP H7N9 strains (GD/17SF003, QY/GIRD1 and GD/17SF006). Red and blue color stripes indicate the overlap time period of estimated divergence time range of HA and NA genes of each virus according to 95% HPD of TMRCA calculated by BEAST 2.0. Abbreviations: CK/J1 (A/Chicken/Guangdong/J1/2017), CK/J2 (A/Chicken/Guangdong/J1/2017), GD/17SF003 (A/Guangdong/17SF003/2016), QY/GIRD1 (A/Qingyuan/GIRD1/2017), GD/17SF006 (A/Guangdong/17SF006/2017), and HPD (highest posterior density).

In agreement with previous findings, during the first and second waves, most viruses in this lineage circulated in the YRD and OR regions^13^. However, from the estimated time from August to October of 2013 (mean TMRCA values of HA and NA genes were, 2013.9618 and 2013.8686, respectively; 95% HPD of HA and NA were 2013.383~2014.000 and 2013.732~2013.900, respectively), a small branch virus began circulating in the PRD region (Figs 3F and 4F). Importantly, all the HA and NA genes of novel HP mutant strains, including human and chicken strains, originated from this branch. The TMRCA of HP mutant strains was estimated at around July 2015 to March 2016 (mean TMRCA values of HA and NA genes were 2016.0987 and 2015.8818, respectively; 95% HPD of HA and NA were 2015.612~2016.561 and 2015.341~2016.279, respectively) (Figs 3F and 4F). According to overlap of the 95% HPD range of HA and NA genes, the two novel AIV strains CK/J1 (mean TMRCA values of HA and NA genes were, 2016.0987 and 2015.8818, respectively; 95% HPD of HA and NA genes were 2015.612~2016.561 and 2015.341~2016.279, respectively) and CK/J2 (mean TMRCA values of HA and NA genes were, 2016.2525 and 2016.0375, respectively; 95% HPD of HA and NA genes were 2015.794~2016.664 and 2015.584~2016.431, respectively) were estimated to appear earlier (the CK/J1 strain was estimated to appear from July 2015 to March 2016, while CK/J2 appeared from September 2015 to May 2016) or had 3 months of overlapped time period (March-May 2016) with the human strain GD/17SF003 (mean TMRCA values of HA and NA genes were, 2016.5776 and 2016.6254, respectively; 95% HPD of HA and NA genes were 2016.107~2016.882 and 2016.312~2016.83, respectively) which was estimated to appear from March to September 2016 (Figs 3F, 4F and 5). According to the molecular clock analysis results of both HA and NA genes, those two novel AIV strains appeared significantly earlier than the human counterparts, such as QY/GIRD1 (mean TMRCA values of HA and NA genes were 2016.9366 and 2016.4983, respectively; 95% HPD of HA and NA genes were 2016.708~2017.000 and 2016.115~2016.769, respectively) and GD/17SF006 (mean TMRCA values of HA and NA genes were 2016.9366 and 2016.6254, respectively; 95% HPD of HA and NA genes were 2016.708~2017.000 and 2016.312~2016.83, respectively), with estimated TMRCA from August to September 2016 (Figs 3F, 4F and 5). Together with the results of molecular clock analysis of HA and NA genes of the novel HP IAV(H7N9), we further confirmed that the avian HP strains appeared months earlier than those human strains during the evolution process.

## Discussion

In this study, the positive rate of potentially HP IAV(H7N9) influenza virus in real time RT-PCR detection was 1% (2/200). Since we only collected samples from one LPM, this finding does not necessarily represent the entire situation of the ongoing 5^th^ epidemic of influenza virus infections in China, but it still reveals a potential threat on public health. In this study, all the sampled birds, including the sources of CK/J1 and CK/J2, showed no significant clinical signs. It is known that occasionally an H5 or H7 virus causes only mild illness in chickens and turkeys, although it has a genetic signature that classifies it as an HPAI virus^14–16^. According to the amino acid signature analysis in our study, this may be related to the non-continuous basic amino acid insertion in the HA cleavage site, different from human HP strains; a further study is required to verify the relationship between such differences and viral pathogenicity^17^. The 226Q and 228G amino acids in the HA protein and/or the 627E and 701D in the PB2 protein indicate that such viruses may still adapt to the avian species^18, 19^. However, these viruses also show some human or mammalian receptor binding characteristics, such as the 138 A, 160 A, 186 V in the HA proteins, indicating their potential adaption to human 2,6-linked sialic acid receptor^20–22^. A study on the recent human HP H7N9 strains GD/17SF003 and GD/17SF006, which both carried the amino acids insertion on HA cleavage site suggested their preference for both avian- and human-type receptors^23^. Considering the LPM plays a critical role in AIV spread to humans, birds with no significant clinical signs may still shed viruses during breeding and growing in farms, transportation and sale in LPMs, which may not be easily noticed. This would increase the risk of AIV spreading to humans and other animals^10^. In such conditions, annual systematic and comprehensive monitoring of IAV(H7N9) should be conducted in LPMs in Guangdong province and potentially elsewhere.

Phylogenetic analysis indicates that genes of the two novel AIVs (CK/J1 and CK/J2) and those of 5 human strains all were reassortant viruses (Fig. 2). The avian strains harbored two surface glycoprotein genes (HA and NA) from PRD clades while the other six internal genes were from the YRD clade (Fig. 2). Different from chicken strains, the human strains in Guangdong province during the fifth wave carried two different genotypes; QY/GIRD1 and GD/17SF006, which contained genes from three clades (PB2, M and NS from YRD; PA, HA, NP and NA from PRD; PB1 from OR). GD/SP440, GD/HP001 and GD/17SF003 contained genes from two clades (PB2, NP, M and NS from YRD; the remaining genes from PRD)^13^. These results indicate that the two main IAV(H7N9) sources were YRD and PRD, corroborating previous findings^13^. In addition, the results suggest that IAV(H7N9) viruses with different genotypes are co-circulating in avian and humans^7, 13^. Moreover, the surface glycoprotein genes, HA and NA of the two avian and five human strains derived from wave 3 are similar to the PRD lineage viruses. According to Wu and colleagues, genes located in such lineage began to circulate in central region of Guangdong province in wave 2^24^. Some genes of the two novel AIVs CK/J1 and CK/J2, such as HA, NA, NP and M, were closely related to human strain lineages^20–22, 25^. However, unlike genes of those 5 human strains, the polymerase tripartite and NS genes of CK/J1 and CK/J2 were closely related to avian strains, indicating that such genes may still circulate and undergo adaptation in poultries of Guangdong.

The molecular clock analysis of the HA and NA genes suggested that the two CK/J1 and CK/J2 strains appeared from July 2015 to March 2016, several months earlier than the newly found human strains with additional cleavage sites in the HA protein, which were estimated to appear in March 2016 (Fig. 5). Such results suggest that the newly identified IAV(H7N9) with HP status may have obtained some of the HP mutations in chicken before adaptation to humans^26^. As shown by the molecular clock study, nine of thirteen viruses in the branch containing the HA gene of novel HP IAV(H7N9) strains were of human sources; only four viruses were avian, including CK/J1 and CK/J2 (Fig. 3F). Taken together, molecular clock analysis, molecular characterization and phylogenetic analyses indicate that the two novel AIV strains may constitute an intermediary virus during IAV(H7N9) spread among avian and humans. However, direct evidence to show that these novel AIVs transmit from avian to humans remains to be demonstrated.

Some limitations of this study are noteworthy. First, because of the necessary biosafety restrictions, we did not attempt to isolate and culture the AIVs collected from positive swab samples. However, since their full genomes have been obtained, these novel AIV can potentially be reconstructed through reverse genetics (e.g., in collaboration with researchers with a BSL-3 lab) for further basic virology research. Secondly, sample collection was carried out in a single day at one LPM; therefore, the results could not be generalized to the whole region and for a longer period of time. Additional surveys with longer time frame and in more sampling sites (e.g., in poultry farms and LPMs in southern China, including but not necessarily limited to Fujian, Hunan, Guangxi and Hainan provinces besides Guangdong province) are being planned. The results from these additional studies will be described in subsequent reports.

In conclusion, we demonstrate for the first time that the IAV(H7N9) viruses isolated from chickens in LPM in Guangdong province have acquired additional basic amino acids in the HA cleavage site, with only one amino acid difference from those found in HP IAV(H7N9) human strains, which can play an important role in increased virulence in humans. According to the divergence time scale, these two AIV strains seemed to have appeared months before the isolation of the human HP strains in Guangdong province. Therefore, the acquired poly-basic insertion in the novel AIVs may be attributed to persistent circulation in poultry species and may serve as intermediary viruses to human infections. Further investigation is required to determine whether the poly-basic HA cleavage site of the IAV(H7N9) virus is associated with increased avian and/or human disease severity. The molecular characteristics of these novel chicken AIV strains highlight challenges in risk assessment of IAV(H7N9) infections at the human-animal interface.

## Materials and Methods

### Ethics Statement

Swab sampling and experiments were approved by the Institutional Animal Care and Use Committee of Guangzhou Medical University. All methods were performed in accordance with the relevant guidelines and regulations.

### Sample collection

A total of 200 tracheal swab samples were collected from chickens, ducks, geese and pigeons, without significant clinical signs, in a LPM located in Guangdong Province, on Feb 25, 2017. Swabs were placed into sterile tubes containing 1 mL of phosphate-buffered saline (PBS) on ice, and transported to the laboratory for further sample processing and RNA extraction within 4 hours. Swabs were vortexed vigorously for 15–20 s and pressed against the tube wall to remove as much organic materials as possible. Then, the samples were cleared by centrifugation for 5 mins at 3000 rpm. The resulting supernatants were stored at −80 °C until use.

### Real time RT-PCR detection and viral genome sequencing

Before testing, samples were thawed at room temperature. Viral RNA was extracted using the RNeasy Minikit (Qiagen, Germany). Real-time reverse transcription-polymerase chain reaction (RT-PCR) was performed with the PCR-Fluorescence Detection Kit for H7 Influenza A virus RNA (Cat No. SJ-LG-004–3, Shanghai Biogerm Biological Technology Co., LTD, Shanghai, China) following the manufacturer’s instructions, on the ABI-7500 Real-time PCR system (Applied Biosystems, Foster City, CA). Ct value ≤ 30 was considered to be positive. One-step RT-PCR was performed on the Bio-Rad T100TM Thermal Cycler (Bio-Rad, Hercules, CA) with the Invitrogen Superscript kit, following the manufacturer’s instructions. The full-length genomic sequences of the two IAV(H7N9) strains were amplified with specific RT-PCR primers. PCR products were separated by 1% agarose gel electrophoresis and purified using the Qiagen gel extraction kit (Qiagen, Inc., Valencia, CA), and cloned into the pMD18-T vector (TaKaRa). The pMD18-T vectors were transformed into DH5α competent cells, and cultured in 37 °C for 20 h. Five colonies of each virus gene segment were sent to Sangon Biotech (Shanghai) Co., Ltd. for complete genome sequencing on the ABI 3730XL automatic DNA analyzer (Applied Biosystems) with the ABI BigDye Terminator v3.1 cycle sequencing kit (Applied Biosystems). None of the work included in this study involved culturing the live viruses. Sample preparation and viral RNA extraction from the swaps were conducted inside a biosafety cabinet in a BSL-2 lab facility following proper biosafety guidelines and procedure approved by the local institution.

### Sequence Alignment and Phylogenetic Analysis

Comparisons of nucleotide and deduced amino acid sequences were performed by Clustal W method using MegAlign 7.0 (DNASTAR Inc, the USA). The initial phylogenetic analysis was conducted, comparing sequences with the genomes of 79 IAV(H7N9) strains (29 avian and 50 human strains) available in Global Initiative on Sharing All Influenza Data (GISAID) (Supplementary Material S1). Phylogenetic trees were generated by the neighbor-joining method using MEGA 7.0 (Molecular Evolutionary Genetics Analysis, USA). Bootstrap values were calculated based on 1000 alignment replicates.

### Molecular Clock Analysis

The time-scale of IAV(H7N9) HA genes (Supplementary Materials S2) evolution from Feb-2013 to Jan-2017 was estimated using the Bayesian Markov chain Monte Carlo (MCMC) method in the BEAST package v1.8.2^27^. A relaxed molecular clock model with uncorrelated log-normally distributed rates (UCLD), the GTR + Gamma + Invariant nucleotides substitution model, and the Bayesian Skyline demographic model were used^28^. Markov chain Monte Carlo (MCMC) algorithm was run for a 100 million step chain and sampled every 10,000 states, and 10% of the chain was removed as burn-in. Times of most common recent ancestor (TMRCAs) on the tree nodes and the 95% highest posterior density (HPD) interval of specific nodes (Figs 3 and 4) were calculated by the BEAST software.

## Electronic supplementary material

Supplementary information
