# Supplementary material for: First genome report and analysis of chicken H7N9 influenza viruses with poly-basic amino acids insertion in the hemagglutinin cleavage site
Source: Sci Rep. 2017 Aug 30;7:9972. doi: 10.1038/s41598-017-10605-6 (PMC5577273; doi:10.1038/s41598-017-10605-6)
Supplement: Supplementary file 1 — Supplementary information [file 41598_2017_10605_MOESM1_ESM.pdf]

First genome report and analysis of chicken H7N9 influenza viruses with poly-basic amino acids insertion in the hemagglutinin cleavage site

Jidang Chen[1,2#], Jipei Zhang[1#], Wanjun Zhu[2], Yishan Zhang[1], Hualong Tan[1], Minfang Liu[1], Mingsheng Cai[3], Jiaren Shen[4], Hinh Ly[2]*, Jianhong Chen[1]*

[1]College of Life Science and Engineering, Foshan University, Foshan, Chin

[2]Department of Veterinary & Biomedical Sciences, College of Veterinary Medicine, University of Minnesota, Twin Cites, Minnesota, USA

[3]Department of Pathogenic Biology and Immunology, Sino-French Hoffmann Institute, School of Basic Medical Science, Guangzhou Medical University, Guangzhou, Guangdong, China

[4]Shanghai Municipal Center For Disease Control And Prevention (SCDC), Shanghai, China

[#]These authors contributed equally to this study.

***Corresponding authors:**

Jianhong Chen

Professor of Preventive Veterinary Medicine

College of Life Science and Engineering, Foshan University, Foshan, China

Tel: +86-0757-85505088

E-mail: jianhongchen.fsu@gmail.com

Hinh Ly

Associate Professor of Virology

University of Minnesota, Twin Cities

1988 Fitch Ave., Ste 295, AS/VM Bldg.

Saint Paul, MN 55108,

Tel: 612-625-3358

Email: hly@umn.edu

Supplementary Table S1. Gene Accession Numbers of H7N9 viruses input for phylogenetic analysis

Strain Name	Data Base	Collected Date	Host	Collected Region	Database Accession No.							
					PB2	PB1	PA	HA	NP	NA	M	NS
A/Chicken/Guangdong/J1/2017[a]	N[f]	2017-02-25	A[b]	PRD[d]	KY855515	KY855516	KY855517	KY855518	KY855519	KY855520	KY855521	KY855522
A/Chicken/Guangdong/J2/2017[a]	N	2017-02-25	A	PRD	KY855523	KY855524	KY855525	KY855526	KY855527	KY855528	KY855529	KY855530
A/Anhui/01876/2014	G[h]	2014-03-10	H[c]	YRD[d]	627398	627399	627397	627401	627394	627400	627396	627395
A/Anhui/09186/2014	G	2014-02-07	H	YRD	628029	628030	628028	628032	628025	628031	628027	628026
A/Anhui/1-YK RG56/2013	G	2015-08-27	H	YRD	637069	637068	635611	637063	637066	637065	637064	637067
A/Anhui/60936/2016	G	2016-12-16	H	YRD	887945	887946	887944	887948	887941	887947	887943	887942
A/Beijing/40610/2015	G	2015-05-26	H	PRD	628541	628542	628540	628544	628537	628543	628539	628538
A/chicken/Dongguan/210/2014	G	2014-02-20	A	PRD	597186	597187	591552	597188	597189	597190	597191	597192
A/chicken/Dongguan/3438/2013	G	2013-12-19	A	PRD	581697	581702	576724	581703	581704	581705	581706	581707
A/chicken/Dongguan/3487/2013	G	2013-12-19	A	PRD	581715	581716	576726	581717	581718	581719	581720	581721
A/chicken/Dongguan/3491/2013	G	2013-12-19	A	PRD	581729	581730	576758	581731	581732	581733	581734	581735
A/chicken/Ganzhou/GZ79/2016	G	2016-01-27	A	PRD	942266	942236	942206	942085	942176	942146	942116	942296
A/Chicken/Guangdong/DG478/2014	G	2014-11-25	A	PRD	580264	580265	580266	580267	580268	580269	580270	580271
A/Chicken/Guangdong/DG593/2014	G	2014-12-12	A	PRD	580296	580297	580298	580299	580300	580301	580304	580305
A/chicken/Guangdong/G1/2013	G	2013-05-05	A	PRD	576596	576590	576560	576566	576578	576572	576602	576584
A/chicken/Guangdong/G2/2013	G	2013-05-05	A	PRD	576597	576591	576561	576567	576579	576573	576603	576585
A/chicken/Guangdong/G3/2013	G	2013-05-05	A	PRD	576598	576592	576562	576568	576580	576574	576604	576586
A/Chicken/Guangdong/SW153/2015	G	2015-02-05	A	PRD	580376	580377	580378	580379	580380	580381	580382	580383
A/chicken/Jiangxi/10882/2014	G	2014-02-18	A	OR[d]	594176	594177	591439	594178	594179	594180	594181	594182
A/chicken/Jiangxi/14517/2014	G	2014-04-20	A	OR	593158	593159	577366	593160	593161	593162	593163	593164
A/chicken/Jiangxi/18482/2014	G	2014-06-15	A	OR	593467	593468	577858	593469	593470	593471	593472	593473
A/chicken/Jiangxi/9558/2014	G	2014-02-16	A	OR	583923	583924	577212	583925	583926	583927	583928	583929
A/Chicken/Jilin/13188/2014	G	2014-02-20	A	OR	457810	457811	457809	528346	457806	528345	528341	528340
A/chicken/Longquan/LQ78/2016	G	2016-01-30	A	YRD	942258	942228	942198	942077	942168	942138	942108	942288
A/chicken/Rizhao/515/2013	G	2013	A	OR	468997	469192	487859	469657	470594	470344	515781	469515

A/chicken/Shanghai/S1078/2013	G	2013-04-03	A	YRD	515775	515776	515777	457813	515779	457812	470073	457807
A/chicken/Shantou/2537/2014	G	2014-04-16	A	PRD	593193	593194	577498	593195	593196	593197	457808	593199
A/chicken/Shenzhen/138/2014	G	2014-02-19	A	PRD	578083	578084	576722	578085	578086	578087	593198	578089
A/chicken/Shenzhen/1665/2013	G	2013-12-12	A	PRD	577950	577951	576689	577952	577953	577954	578088	577956
A/chicken/Wenzhou/WZTSLG02/2015	G	2015-01	A	YRD	683200	683160	683245	682924	683072	683031	577955	683113
A/chicken/Zhangzhou/8585/2014	G	2014-06-23	A	OR	593643	593644	591402	593645	593646	593647	682985	593649
A/chicken/Zhejiang/DTID-ZJU06/2013	G	2013-12	A	YRD	590923	590924	590925	590926	590927	590928	593648	590930
A/Duck/Guangdong/DG103/2015	G	2015-01-07	A	PRD	580324	580325	580326	580327	580328	580329	590929	580331
A/Fujian/02152/2017	G	2017-01-06	H	OR	887617	887618	887616	887620	887613	887619	580330	887614
A/Fujian/1/2016	G	2016-01-13	H	OR	833701	833702	833704	833705	833706	833707	887615	833709
A/Fujian/2/2015	G	2015-01-03	H	OR	627462	627463	627461	627465	627458	627464	833708	627459
A/Fujian/21/2014	G	2014-12-29	H	OR	627374	627375	627373	627377	627370	627376	627460	627371
A/Fujian/54840/2016	G	2016-11-16	H	OR	888081	888082	888080	888084	888077	888083	627372	888078
A/GD-120/2015/H7N9/2015-03-10	G	2015-03-10	H	PRD	656503	656504	656502	656506	656499	656505	888079	656500
A/GD-153/2014/H7N9/2014-05-08	G	2014-05-08	H	PRD	656239	656240	656238	656242	656235	656241	656501	656236
A/Guangdong/036/2014	G	2014-01-13	H	PRD	628461	628462	628460	628464	628457	628463	656237	628458
A/Guangdong/15SF043/2015	G	2015-01-23	H	PRD	627110	627111	627109	627113	627106	627112	628459	627107
A/Guangdong/17SF003/2016	G	2017-01-03	H	PRD	919596	919597	919595	919607	919592	919606	627108	919601
A/Guangdong/17SF006/2017	G	2017-01-12	H	PRD	509127	509126	509125	919599	509123	919598	919602	919593
A/Guangdong/2/2013	G	2013	H	PRD	-[e]	-	-	499628	-	503535	-	-
A/Guangdong/24997/2013	G	2013-11-30	H	PRD	628517	628518	628516	628520	628513	628519	628515	628514
A/Guangdong/24999/2013	G	2013-12-16	H	PRD	628509	628510	628508	628512	628505	628511	628507	628506
A/Guangdong/HP001/2017	G	2017-01-04	H	PRD	960354	960356	960358	960360	960362	960364	960366	960368
A/Guangdong/SP440/2017	G	2017-01-04	H	PRD	960355	960357	960359	960361	960363	960365	960367	960369
A/Guangdong-Guangzhou/XN00457/2014	G	2014-02-07	H	PRD	627750	627751	627749	627753	627746	627752	627748	627747
A/Guangdong-Guangzhou/XN00588/2014	G	2014-02-16	H	PRD	627654	627655	627653	627657	627650	627656	627652	627651
A/Hebei/01/2013	G	2013-07-19	H	OR	447865	447863	447862	509120	447858	509122	509121	509124
A/Hubei/34007/2015	G	2015-04-22	H	OR	628613	628614	628612	628616	628609	628615	628611	628610

A/Huizhou/01/2013	G	2013-08-08	H	PRD	503496	503497	503518	503499	503500	503501	503502	503503
A/Hunan/02285/2017	G	2017-01-08	H	OR	887641	887642	887640	887644	887637	887643	887639	887638
A/Hunan/26937/2014	G	2014-04-19	H	OR	627845	627846	627844	627848	627841	627847	627843	627842
A/jiangsu/03/2013	G	2013-04-06	H	YRD	439488	439489	439490	447602	439491	447857	447855	447860
A/Jiangsu/60456/2016	G	2016-12-02	H	YRD	887929	887930	887928	887932	887925	887931	887927	887926
A/Jiangsu/60457/2016	G	2016-12-06	H	YRD	887921	887922	887920	887924	887917	887923	887919	887918
A/Jiangsu/60466/2016	G	2016-11-21	H	YRD	887825	887826	887824	887828	887821	887827	887823	887822
A/Nanchang/1/2013	G	2013-04-24	H	OR	453601	453602	453620	460535	453605	467774	461480	461481
A/Nanjing/1/2013	G	2013-03-28	H	YRD	919604	919605	919603	453604	919600	453606	453607	453608
A/Nanjing/5/2013	G	2013-04-25	H	YRD	460772	460773	460774	460775	460776	460777	460778	460779
A/Qingyuan/GIRD1/2017	G	2017-01-14	H	PRD	918730	918732	918734	918736	918737	918738	918739	918740
A/Quzhou/1/2015	G	2015-04-25	H	YRD	621126	621125	621113	621120	621123	621121	621122	621124
A/Shanghai/1/2013	G	2013-02-26	H	YRD	528343	528344	528342	439486	528339	439487	439493	439494
A/Shanghai/Patient6/2013	G	2013-05-01	H	YRD	451269	451270	451271	451272	451273	451274	451275	451276
A/silkie chicken/Dongguan/3606/2013	G	2013-12-19	A	PRD	581836	581837	577199	581838	581839	581840	581841	581870
A/silkie chicken/Dongguan/656/2014	G	2014-02-20	A	PRD	596828	596829	591518	596830	596831	596832	596833	-
A/silkie chicken/Jiangxi/9476/2014	G	2014-02-16	A	YRD	583903	583904	577202	583905	583906	583907	583908	583950
A/Xinjiang/73030/2014	G	2014-07-25	H	OR	627917	627918	627916	627920	627913	627919	627915	627914
A/Xinjiang/75802/2014	G	2014-08-15	H	OR	627829	627830	627828	627832	627825	627831	627827	627826
A/XinjiangBintuan/99117/2014	G	2014-10-25	H	OR	566097	566098	566096	566100	566093	566099	566095	566094
A/XinjiangBintuan/99118/2014	G	2014-11-20	H	OR	566105	566106	566104	566108	566101	566107	566103	566102
A/Zhejiang/1/2017	G	2017-01-07	H	YRD	887721	887722	887720	887724	887717	887723	887719	887718
A/Zhejiang/19/2016	G	2016-12-28	H	YRD	887673	887674	887672	887676	887669	887675	887671	887670
A/Zhejiang/22/2013	G	2013-10-14	H	YRD	477407	477408	477409	477410	477411	477412	477413	477414
A/Zhejiang/5/2017	G	2017-01-02	H	YRD	887657	887658	887656	887660	887653	887659	887655	887654
A/Zhejiang/6/2016	G	2016-10-07	H	YRD	887801	887802	887800	887804	887797	887803	887799	887798
A/Zhejiang/6/2017	G	2017-01-05	H	YRD	887649	887650	887648	887652	887645	887651	887647	887646
A/Zhejiang/8/2015	G	2015-02-02	H	YRD	628765	628766	628764	628768	628761	628767	628763	628762

Note

a. The accession numbers of A/Chicken/Guangdong/J1/2017 and A/Chicken/Guangdong/J2/2017 were assigned by National Center of Biology Information (NCBI), the accession number of the rest strains were assigned by Global Initiative on Sharing All Influenza Data (GISAID). The first 3 characters "EPI" of all the accession number assigned by GISAID were omitted in the table.

b. Letter "A" represent "Avian"

c. Letter "H" represent "Human"

d. Abbreviation: "YRD" represent Yangtze River Delta, "PRD" represent Pearl River Delta and OR represent other regions.

e. "-" indicate the sequence information was not available in both GISAID or NCBI, and the indicated sequences were not included in the phylogenetic trees calculation.

f. Letter "N" represent "NCBI" database.

h. Letter "G" represent "GISAID" database.

Supplementary Table S2. HA and NA gene Accession Numbers of H7N9 viruses input for molecular clock analysis

Strain Name	Data Base	Isolated Date	Database Accession No. HA	NA	Host	Isolated Region
A/Anhui/1-YK RG182/2013	G	2013	637962	637964	Human	YRD
A/chicken/Henan/102/2013	G	2013	692155	691756	Avian	OR
A/chicken/Henan/115/2013	G	2013	692162	691763	Avian	OR
A/chicken/Henan/120/2013	G	2013	692164	691765	Avian	OR
A/chicken/Henan/141/2013	G	2013	692165	691766	Avian	OR
A/Shanghai/1/2013	G	2013-02-26	439486	439487	Human	YRD
A/Minhang/S01/2013	G	2013-02-27	941039	941041	Human	YRD
A/Shanghai/3/2013	G	2013-02-27	443022	443023	Human	YRD
A/Shanghai/02/2013	G	2013-03-05	568648	448938	Human	YRD
A/Shanghai/4664T/2013	G	2013-03-05	446962	446965	Human	YRD
A/Zhejiang/1/2013	G	2013-03-24	443034	443036	Human	YRD
A/chicken/Anhui-Chuzhou/01/2013	G	2013-03-29	447600	447884	Avian	YRD
A/Wuxi/2/2013	G	2013-03-31	467313	467315	Human	YRD
A/chicken/Jiangsu/1021/2013	G	2013-04	499887	499886	Avian	YRD
A/chicken/Zhejiang/C483/2013	G	2013-04	532303	532271	Avian	YRD
A/chicken/Zhejiang/DTID-ZJU01/2013	G	2013-04	442721	442723	Avian	YRD
A/Zhejiang/32/2013	G	2013-04	476480	-	Human	YRD
A/Anhui/04/2013	G	2013-04-01	509086	509084	Human	YRD
A/pigeon/Shanghai/S1421/2013	G	2013-04-03	457637	457636	Avian	YRD
A/Shanghai/ION/2013	G	2013-04-04	585979	585980	Human	YRD
A/chicken/Wuxi/0405005/2013	G	2013-04-05	663437	663451	Avian	YRD
A/chicken/Jiangxi/12544/2013	G	2013-04-07	583207	583209	Avian	OR
A/chicken/Jiangxi/12564/2013	G	2013-04-07	591857	591859	Avian	OR
A/Chicken/Nanjing/761/2013	G	2013-04-09	515788	515790	Avian	YRD
A/Suzhou/3/2013	G	2013-04-09	497894	497896	Human	YRD
A/Nanjing/6/2013	G	2013-04-11	497902	497904	Human	YRD
A/Suzhou/5/2013	G	2013-04-12	497910	497912	Human	YRD
A/Nanjing/7/2013	G	2013-04-13	497918	497920	Human	YRD
A/chicken/Jiangsu/SC035/2013	G	2013-04-16	457869	457868	Avian	YRD
A/chicken/Jiangsu/SC537/2013	G	2013-04-16	457853	457852	Avian	YRD
A/Hangzhou/254/2013	G	2013-04-17	450842	450844	Human	YRD
A/Shanghai/MH01/2013	G	2013-04-17	542311	542313	Human	YRD
A/wild pigeon/Jiangsu/SD001/2013	G	2013-04-17	457621	457620	Avian	YRD
A/Anhui/03/2013	G	2013-04-21	447630	447632	Human	YRD
A/chicken/Zhejiang/SD007/2013	G	2013-04-22	457765	457764	Avian	YRD
A/Jiangsu/Wuxi04/2013	G	2013-04-26	756011	756016	Human	YRD
A/Fujian/02/2013	G	2013-04-27	509087	509089	Human	OR
A/Fujian/05/2013	G	2013-04-27	509118	509119	Human	OR
A/Fujian/03/2013	G	2013-04-29	509102	509100	Human	OR
A/Fujian/04/2013	G	2013-05-02	509103	509105	Human	OR
A/chicken/Guangdong/SD641/2013	G	2013-05-03	457885	457884	Avian	PRD

A/chicken/Jiangxi/SD001/2013	G	2013-05-03	457845	457844	Avian	OR
A/chicken/Guangdong/G1/2013	G	2013-05-05	576566	576572	Avian	PRD
A/chicken/Guangdong/G3/2013	G	2013-05-05	576568	576574	Avian	PRD
A/chicken/Hangzhou/174/2013	G	2013-05-05	639587	639589	Avian	YRD
A/tree sparrow/Shanghai/01/2013	G	2013-05-09	508926	507149	Avian	YRD
A/chicken/Guangdong/3640/2013	G	2013-05-16	576565	576571	Avian	PRD
A/chicken/Guangdong/SD1/2013	G	2013-05-16	576569	576575	Avian	PRD
A/chicken/Guangzhou/1/2013	G	2013-07-12	508781	-	Avian	PRD
A/Hebei/01/2013	G	2013-07-19	509120	509122	Human	OR
A/Huizhou/01/2013	G	2013-08-08	503499	503501	Human	PRD
A/chicken/Shaoxing/2417/2013	G	2013-10-20	582340	582342	Avian	YRD
A/chicken/Huzhou/3791/2013	G	2013-10-24	582354	582356	Avian	OR
A/chicken/Huzhou/3802/2013	G	2013-10-24	582361	582907	Avian	OR
A/chicken/Huzhou/4067/2013	G	2013-10-24	582944	582946	Avian	OR
A/chicken/Huzhou/4074/2013	G	2013-10-24	582966	582968	Avian	OR
A/chicken/Huzhou/4076/2013	G	2013-10-24	582983	582985	Avian	OR
A/silkie chicken/Huzhou/4213/2013	G	2013-10-24	583044	583046	Avian	OR
A/chicken/Jiaxing/4490/2013	G	2013-10-25	583072	583074	Avian	OR
A/chicken/Shaoxing/5087/2013	G	2013-10-28	583095	583097	Avian	YRD
A/chicken/Shaoxing/5201/2013	G	2013-10-28	583130	583132	Avian	YRD
A/chicken/Shaoxing/5227/2013	G	2013-10-28	583144	583146	Avian	YRD
A/silkie chicken/Shaoxing/5130/2013	G	2013-10-28	583102	583104	Avian	YRD
A/silkie chicken/Shaoxing/5235/2013	G	2013-10-28	583151	583153	Avian	YRD
A/chicken/Shaoxing/5479/2013	G	2013-10-29	583166	583168	Avian	YRD
A/Guangdong/DG-02/2013	G	2013-11	515861	515860	Human	PRD
A/Guangdong/24997/2013	G	2013-11-30	628520	628519	Human	PRD
A/Guangdong/DG-03/2013	G	2013-12	515869	515868	Human	PRD
A/Guangdong/YJ-04/2013	G	2013-12	515877	515876	Human	PRD
A/Guangdong/YJ-05/2013	G	2013-12	515885	515884	Human	PRD
A/chicken/Shenzhen/2293/2013	G	2013-12-13	581595	581597	Avian	PRD
A/Guangdong/24999/2013	G	2013-12-16	628512	628511	Human	PRD
A/chicken/Dongguan/3141/2013	G	2013-12-18	581622	581624	Avian	PRD
A/chicken/Dongguan/3145/2013	G	2013-12-18	581629	581631	Avian	PRD
A/silkie chicken/Dongguan/3049/2013	G	2013-12-18	581609	581611	Avian	PRD
A/chicken/Dongguan/4040/2013	G	2013-12-19	583318	583320	Avian	PRD
A/chicken/Dongguan/4064/2013	G	2013-12-19	583311	583313	Avian	PRD
A/chicken/Dongguan/4251/2013	G	2013-12-19	582325	582327	Avian	PRD
A/chicken/Shenzhen/3733/2013	G	2013-12-19	581844	581846	Avian	PRD
A/chicken/Shenzhen/3734/2013	G	2013-12-19	581851	581853	Avian	PRD
A/silkie chicken/Dongguan/3605/2013	G	2013-12-19	581832	581834	Avian	PRD
A/silkie chicken/Dongguan/3606/2013	G	2013-12-19	581838	581840	Avian	PRD
A/silkie chicken/Shenzhen/3781/2013	G	2013-12-19	581865	581867	Avian	PRD
A/silkie chicken/Shenzhen/3782/2013	G	2013-12-19	581873	581875	Avian	PRD
A/Shanghai/01/2014	G	2014-01-03	531468	531470	Human	YRD

A/Guangdong/02620/2014	G	2014-01-08	628504	628503	Human	PRD
A/chicken/China/028/2014	G	2014-01-10	703994	703996	Avian	NA
A/Guangdong/035/2014	G	2014-01-13	628472	628471	Human	PRD
A/Hunan/08963/2014	G	2014-02-02	628296	628295	Human	OR
A/Hunan/09193/2014	G	2014-02-05	628184	628183	Human	OR
A/Anhui/09186/2014	G	2014-02-07	628032	628031	Human	YRD
A/Huaian/062/2014	N	2014-02-14	KP864455	KP864449	Human	YRD
A/chicken/Jiangxi/9534/2014	G	2014-02-16	583918	583920	Avian	OR
A/chicken/Jiangxi/10885/2014	G	2014-02-18	594185	594187	Avian	OR
A/chicken/Jiangxi/10896/2014	G	2014-02-18	594206	594208	Avian	OR
A/chicken/Jiangxi/10964/2014	G	2014-02-18	594346	594348	Avian	OR
A/chicken/Dongguan/864/2014	G	2014-02-20	597352	597354	Avian	PRD
A/chicken/Dongguan/1124/2014	G	2014-02-21	597453	597455	Avian	PRD
A/chicken/Dongguan/1177/2014	G	2014-02-21	596947	596949	Avian	PRD
A/chicken/Dongguan/1307/2014	G	2014-02-21	597512	597514	Avian	PRD
A/chicken/Dongguan/1314/2014	G	2014-02-21	597519	597521	Avian	PRD
A/chicken/Dongguan/1382/2014	G	2014-02-21	597551	597553	Avian	PRD
A/chicken/Dongguan/1421/2014	G	2014-02-21	597584	597586	Avian	PRD
A/chicken/Dongguan/1533/2014	G	2014-02-21	578178	578180	Avian	PRD
A/chicken/Dongguan/1697/2014	G	2014-02-21	578224	578226	Avian	PRD
A/silkie chicken/Dongguan/1641/2014	G	2014-02-21	578192	578194	Avian	PRD
A/Huaian/065/2014	N	2014-02-24	KP864457	KP864458	Human	YRD
A/Huaian/074/2014	N	2014-03-02	KP864459	KP864446	Human	YRD
A/chicken/Jiangxi/12200/2014	G	2014-03-16	592172	592174	Avian	OR
A/chicken/Jiangxi/12208/2014	G	2014-03-16	592333	592335	Avian	OR
A/chicken/Jiangxi/12219/2014	G	2014-03-16	592347	592349	Avian	OR
A/chicken/Jiangxi/12245/2014	G	2014-03-16	592059	592061	Avian	OR
A/chicken/Jiangxi/12249/2014	G	2014-03-16	592066	592068	Avian	OR
A/chicken/Jiangxi/12251/2014	G	2014-03-16	592396	592398	Avian	OR
A/chicken/Jiangxi/12261/2014	G	2014-03-16	592080	592082	Avian	OR
A/chicken/Jiangxi/12264/2014	G	2014-03-16	592418	592420	Avian	OR
A/Shantou/1002/2014	G	2014-03-23	531777	531776	Human	PRD
A/Shenzhen/SP126/2014	G	2014-03-23	592186	592188	Human	PRD
A/Shenzhen/SP139/2014	G	2014-04-02	592193	592195	Human	PRD
A/chicken/Jiangxi/13507/2014	G	2014-04-06	592476	592478	Avian	OR
A/chicken/Jiangxi/13524/2014	G	2014-04-06	592515	592517	Avian	OR
A/chicken/Jiangxi/13543/2014	G	2014-04-06	592577	592579	Avian	OR
A/chicken/Jiangxi/13548/2014	G	2014-04-06	592592	592594	Avian	OR
A/chicken/Jiangxi/13502/2014	G	2014-04-11	592230	592232	Avian	OR
A/chicken/Jiangxi/13510/2014	G	2014-04-11	592237	592239	Avian	OR
A/chicken/Jiangxi/13553/2014	G	2014-04-11	592288	592290	Avian	OR
A/Anhui/01881/2014	G	2014-04-13	627417	627416	Human	YRD
A/chicken/Jiangxi/14033/2014	G	2014-04-13	592639	592659	Avian	OR
A/Hunan/26938/2014	G	2014-04-19	627856	627855	Human	OR

A/chicken/Jiangxi/14517/2014	G	2014-04-20	593160	593162	Avian	OR
A/chicken/Jiangxi/14518/2014	G	2014-04-20	593167	593169	Avian	OR
A/chicken/Jiangxi/15524/2014	G	2014-05-05	593278	593280	Avian	OR
A/Anhui/01887/2014	G	2014-05-20	627441	627440	Human	YRD
A/Shandong/01/2014	G	2014-05-22	627808	627807	Human	YRD
A/Huaian/083/2014	N	2014-05-27	KP864443	KP864441	Human	YRD
A/chicken/Jiangxi/18008/2014	G	2014-06-07	593381	593383	Avian	OR
A/chicken/Jiangxi/18482/2014	G	2014-06-15	593469	593471	Avian	OR
A/chicken/Jiangxi/18487/2014	G	2014-06-15	593497	593499	Avian	OR
A/chicken/Jiangxi/18513/2014	G	2014-06-15	593541	593543	Avian	OR
A/chicken/Zhangzhou/8585/2014	G	2014-06-23	593645	593647	Avian	OR
A/chicken/Shantou/4325/2014	G	2014-07-01	593319	593321	Avian	PRD
A/chicken/Shantou/4824/2014	G	2014-07-22	593341	593343	Avian	PRD
A/Xinjiang/73030/2014	G	2014-07-25	627920	627919	Human	OR
A/Xinjiang/75802/2014	G	2014-08-15	627832	627831	Human	OR
A/XinjiangBintuan/99117/2014	G	2014-10-25	566100	566099	Human	OR
A/Chicken/Guangdong/DG478/2014	G	2014-11-25	580267	580269	Avian	PRD
A/Xinjiang/98692/2014	G	2014-11-25	566116	566115	Human	OR
A/Zhejiang/17/2014	G	2014-11-25	552399	552401	Human	YRD
A/Guangdong/02496/2014	G	2014-11-27	566092	566091	Human	PRD
A/Chicken/Guangdong/DG479/2014	G	2014-11-30	580275	580277	Avian	PRD
A/Duck/Guangdong/DG527/2014	G	2014-12-02	580283	580285	Avian	PRD
A/Fujian/17/2014	G	2014-12-02	627481	627480	Human	OR
A/Guangdong/02497/2014	G	2014-12-03	627385	627384	Human	PRD
A/Chicken/Guangdong/DG592/2014	G	2014-12-08	580291	580293	Avian	PRD
A/Chicken/Guangdong/DG593/2014	G	2014-12-12	580299	580301	Avian	PRD
A/Xinjiang/05916/2014	G	2014-12-25	566052	566051	Human	OR
A/Zhejiang/36/2014	G	2014-12-28	627129	627128	Human	YRD
A/Fujian/22/2014	G	2014-12-29	627369	627368	Human	OR
A/Jiangsu/06307/2014	G	2014-12-29	566138	566137	Human	YRD
A/chicken/Taizhou/TZJF02/2015	G	2015-01	682921	683026	Avian	YRD
A/chicken/Wenzhou/HATSLG01/2015	G	2015-01	682923	683029	Avian	YRD
A/chicken/Wenzhou/RAQL01/2015	G	2015-01	682918	683027	Avian	YRD
A/chicken/Wenzhou/RAQL18/2015	G	2015-01	682919	683028	Avian	YRD
A/chicken/Wenzhou/WZTSLG02/2015	G	2015-01	682924	683031	Avian	YRD
A/duck/Wenzhou/RAQL10/2015	G	2015-01	682920	683025	Avian	YRD
A/duck/Wenzhou/YJYF24/2015	G	2015-01	682922	683030	Avian	YRD
A/Chicken/Guangdong/GZ068/2015	G	2015-01-01	580363	580365	Avian	PRD
A/Chicken/Guangdong/HZ098/2015	G	2015-01-01	580313	580317	Avian	PRD
A/Fujian/1/2015	G	2015-01-01	627473	627472	Human	OR
A/Guangdong/15SF002/2015	G	2015-01-05	627209	627208	Human	PRD
A/Duck/Guangdong/DG103/2015	G	2015-01-07	580327	580329	Avian	PRD
A/Fujian/9/2015	G	2015-01-13	627281	627280	Human	OR
A/Guangdong/15SF020/2015	G	2015-01-13	627193	627192	Human	PRD

A/Chicken/Guangdong/DG120/2015	G	2015-01-15	580347	580349	Avian	PRD
A/Fujian/17/2015	G	2015-01-15	627105	627104	Human	OR
A/Jiangsu/22184/2015	G	2015-01-16	626985	626984	Human	YRD
A/Chicken/Guangdong/CZ145/2015	G	2015-02-01	580371	580373	Avian	PRD
A/Zhejiang/8/2015	G	2015-02-02	628768	628767	Human	YRD
A/Huaian/002/2015	N	2015-02-03	KP864460	KP864444	Human	YRD
A/Guangdong/15SF053/2015	G	2015-02-04	627065	627064	Human	PRD
A/Zhejiang/7/2015	G	2015-02-04	628776	628775	Human	YRD
A/Chicken/Guangdong/SW153/2015	G	2015-02-05	580379	580381	Avian	PRD
A/chicken/Huaian/003/2015	N	2015-02-05	KP864442	KP864454	Avian	YRD
A/Guangdong/15SF082/2015	G	2015-02-06	626993	626992	Human	PRD
A/Chicken/Guangdong/SW154/2015	G	2015-02-10	580387	580389	Avian	PRD
A/Guangdong/15SF080/2015	G	2015-02-10	627001	627000	Human	PRD
A/Guangdong/15SF081/2015	G	2015-02-10	627049	627048	Human	PRD
A/Hunan/19762/2015	G	2015-02-10	627081	627080	Human	OR
A/chicken/Quzhou/2/2015	G	2015-02-24	621115	621116	Avian	YRD
A/Zhejiang/11/2015	G	2015-03-03	628816	628815	Human	YRD
A/Anhui/33224/2015	G	2015-03-05	628744	628743	Human	YRD
A/Shandong-Taian/01/2015	G	2015-03-07	628784	628783	Human	YRD
A/Anhui/33228/2015	G	2015-04-08	628728	628727	Human	YRD
A/Hubei/34007/2015	G	2015-04-22	628616	628615	Human	OR
A/Quzhou/2/2015	G	2015-04-23	621127	621128	Human	YRD
A/Quzhou/1/2015	G	2015-04-25	621120	621121	Human	YRD
A/Anhui/40094/2015	G	2015-05-23	628560	628559	Human	YRD
A/Anhui/40095/2015	G	2015-05-23	628568	628567	Human	YRD
A/Beijing/40610/2015	G	2015-05-26	628544	628543	Human	OR
A/Anhui/1-YK RG56/2013	G	2015-08-27	637063	637065	Human	YRD
A/Fujian/1/2016	G	2016-01-13	833705	833707	Human	OR
A/Fujian/3/2016	G	2016-01-15	833714	833716	Human	OR
A/chicken/Ganzhou/GZ79/2016	G	2016-01-27	942085	942146	Avian	OR
A/chicken/Longquan/LQ78/2016	G	2016-01-30	942077	942138	Avian	YRD
A/Zhejiang/6/2016	G	2016-10-07	887804	887803	Human	YRD
A/Jiangsu/60467/2016	G	2016-10-26	887860	887859	Human	YRD
A/Fujian/54840/2016	G	2016-11-16	888084	888083	Human	OR
A/Fujian/56600/2016	G	2016-11-20	887852	887851	Human	OR
A/Jiangsu/60466/2016	G	2016-11-21	887828	887827	Human	YRD
A/Jiangsu/60456/2016	G	2016-12-02	887932	887931	Human	YRD
A/Zhejiang/7/2016	G	2016-12-05	887708	887707	Human	YRD
A/Jiangsu/60457/2016	G	2016-12-06	887924	887923	Human	YRD
A/Jiangsu/60465/2016	G	2016-12-09	887868	887867	Human	YRD
A/Jiangsu/60458/2016	G	2016-12-10	887916	887915	Human	YRD
A/Jiangsu/60459/2016	G	2016-12-12	887908	887907	Human	YRD
A/Guangdong/60061/2016	G	2016-12-13	887980	887979	Human	PRD
A/Anhui/60928/2016	G	2016-12-15	888012	888011	Human	YRD

A/Jiangsu/60462/2016	G	2016-12-16	887884	887883	Human	YRD
A/Anhui/60927/2016	G	2016-12-18	887988	887987	Human	YRD
A/Zhejiang/10/2016	G	2016-12-18	887780	887779	Human	YRD
A/Anhui/60935/2016	G	2016-12-19	888060	888059	Human	YRD
A/Jiangsu/60450/2016	G	2016-12-19	887964	887963	Human	YRD
A/Jiangsu/60451/2016	G	2016-12-19	887956	887955	Human	YRD
A/Jiangsu/60461/2016	G	2016-12-20	887892	887891	Human	YRD
A/Anhui/60926/2016	G	2016-12-21	888076	888075	Human	YRD
A/Zhejiang/15/2016	G	2016-12-21	887740	887739	Human	YRD
A/Zhejiang/13/2016	G	2016-12-24	887756	887755	Human	YRD
A/Zhejiang/12/2016	G	2016-12-25	887764	887763	Human	YRD
A/Zhejiang/18/2016	G	2016-12-27	887692	887691	Human	YRD
A/Guangdong/60923/2016	G	2016-12-28	887836	887835	Human	PRD
A/Zhejiang/20/2016	G	2016-12-30	887668	887667	Human	YRD
A/Zhejiang/17/2016	G	2016-12-31	887700	887699	Human	YRD
A/Fujian/02151/2017	G	2017-01-01	887612	887611	Human	OR
A/Zhejiang/3/2017	G	2017-01-01	887812	887811	Human	YRD
A/Zhejiang/5/2017	G	2017-01-02	887660	887659	Human	YRD
A/Guangdong/17SF003/2016	G	2017-01-03	919607	919606	Human	PRD
A/Zhejiang/2/2017	G	2017-01-04	887716	887715	Human	YRD
A/Zhejiang/4/2017	G	2017-01-05	887684	887683	Human	YRD
A/Zhejiang/6/2017	G	2017-01-05	887652	887651	Human	YRD
A/Fujian/02152/2017	G	2017-01-06	887620	887619	Human	OR
A/Hunan/02286/2017	G	2017-01-06	887628	887627	Human	OR
A/Zhejiang/1/2017	G	2017-01-07	887724	887723	Human	YRD
A/Hunan/02285/2017	G	2017-01-08	887644	887643	Human	OR
A/Hunan/02287/2017	G	2017-01-11	887636	887635	Human	OR
A/Guangdong/17SF006/2017	G	2017-01-12	919599	919598	Human	PRD
A/Qingyuan/GIRD1/2017	G	2017-01-14	918736	918738	Human	PRD

Note: the accession number of the strains showed in this table were assigned by Global Initiative on Sharing All Influenza Data (GISAID) or National Center of Biology Information (NCBI). The first 3 characters "EPI" of all the accession number assigned by GISAID were omitted in the table. "-" indicate the sequence information was not available in both GISAID or NCBI, and the indicated sequences were not included in the phylogenetic trees calculation.

Abbreviation: "YRD" represent Yangtze River Delta, "PRD" represent Pearl River Delta and OR represent other regions. Letter "G" and "N" in the "Database" column represent "GISAID" and "NCBI", respectively.

Supplementary Table S3. GISAID Influenza Data Acknowledgment

We acknowledge those who contributed to the generation of the genome sequences of influenza A (H7N9) viruses in GISAID, for which this research is based. The list is detailed below.

Isolate name	Isolate ID	Segments	Country	Submitting/Originating Lab	Submmiter
A/Anhui/01876/2014	EPI ISL 192326	8 segments	China	WHO Chinese National Influenza Center	Yang Lei
A/Anhui/09186/2014	EPI ISL 192405	8 segments	China	WHO Chinese National Influenza Center	Yang Lei
A/Anhui/1-YK RG56/2013	EPI ISL 194320	8 segments	China	Other Database Import	EpiFluDB
A/Anhui/60936/2016	EPI ISL 242884	8 segments	China	WHO Chinese National Influenza Center	Yang Lei
A/Beijing/40610/2015	EPI ISL 192469	8 segments	China	WHO Chinese National Influenza Center	Yang Lei
A/chicken/Dongguan/210/2014	EPI ISL 176282	8 segments	China	Other Database Import	EpiFluDB
A/chicken/Dongguan/3438/2013	EPI ISL 175674	8 segments	China	Other Database Import	EpiFluDB
A/chicken/Dongguan/3487/2013	EPI ISL 175676	8 segments	China	Other Database Import	EpiFluDB
A/chicken/Dongguan/3491/2013	EPI ISL 175678	8 segments	China	Other Database Import	EpiFluDB
A/chicken/Ganzhou/GZ79/2016	EPI ISL 252837	8 segments	China	Other Database Import	EpiFluDB
A/Chicken/Guangdong/DG478/2014	EPI ISL 176816	8 segments	China	South China Agricultural University	Weixin Jia
A/Chicken/Guangdong/DG593/2014	EPI ISL 176820	8 segments	China	South China Agricultural University	Weixin Jia
A/chicken/Guangdong/G1/2013	EPI ISL 175606	8 segments	China	Other Database Import	EpiFluDB
A/chicken/Guangdong/G2/2013	EPI ISL 175607	8 segments	China	Other Database Import	EpiFluDB
A/chicken/Guangdong/G3/2013	EPI ISL 175608	8 segments	China	Other Database Import	EpiFluDB
A/Chicken/Guangdong/SW153/2015	EPI ISL 176836	8 segments	China	South China Agricultural University	Weixin Jia
A/chicken/Jiangxi/10882/2014	EPI ISL 176046	8 segments	China	Other Database Import	EpiFluDB
A/chicken/Jiangxi/14517/2014	EPI ISL 180036	8 segments	China	Other Database Import	EpiFluDB
A/chicken/Jiangxi/18482/2014	EPI ISL 180141	8 segments	China	Other Database Import	EpiFluDB
A/chicken/Jiangxi/9558/2014	EPI ISL 176036	8 segments	China	Other Database Import	EpiFluDB
A/Chicken/Jilin/13188/2014	EPI ISL 161671	8 segments	China	WHO Chinese National Influenza Center	Yang Lei
A/chicken/Longquan/LQ78/2016	EPI ISL 252829	8 segments	China	Other Database Import	EpiFluDB
A/chicken/Rizhao/515/2013	EPI ISL 146183	8 segments	China	Other Database Import	EpiFluDB
A/chicken/Shanghai/S1078/2013	EPI ISL 142925	8 segments	China	Harbin Veterinary Research Institute (CAAS)	Kong Huihui
A/chicken/Shantou/2537/2014	EPI ISL 180073	8 segments	China	Other Database Import	EpiFluDB

A/chicken/Shenzhen/138/2014	EPI ISL 176419	8 segments	China	Other Database Import	EpiFluDB
A/chicken/Shenzhen/1665/2013	EPI ISL 175641	8 segments	China	Other Database Import	EpiFluDB
A/chicken/Wenzhou/WZTSLG02/2015	EPI ISL 205128	8 segments	China	Other Database Import	EpiFluDB
A/chicken/Zhangzhou/8585/2014	EPI ISL 180156	8 segments	China	Other Database Import	EpiFluDB
A/chicken/Zhejiang/DTID-ZJU06/2013	EPI ISL 179671	8 segments	China	Other Database Import	EpiFluDB
A/Duck/Guangdong/DG103/2015	EPI ISL 176828	8 segments	China	South China Agricultural University	Weixin Jia
A/Fujian/02151/2017	EPI ISL 242842	8 segments	China	WHO Chinese National Influenza Center / Fujian Provincial Center for Disease Control and Prevention	Yang Lei
A/Fujian/1/2016	EPI ISL 233628	8 segments	China	Fujian Center for Disease Control and Prevention	Jianfeng Xie
A/Fujian/2/2015	EPI ISL 192334	8 segments	China	WHO Chinese National Influenza Center	Yang Lei
A/Fujian/21/2014	EPI ISL 192323	8 segments	China	WHO Chinese National Influenza Center	Yang Lei
A/Fujian/54840/2016	EPI ISL 242901	8 segments	China	WHO Chinese National Influenza Center	Yang Lei
A/GD-120/2015/H7N9/2015-03-10	EPI ISL 198750	8 segments	China	Guangdong Provincial Center for Disease Control and Prevention	Lu Jing
A/GD-153/2014/H7N9/2014-05-08	EPI ISL 198717	8 segments	China	Guangdong Provincial Center for Disease Control and Prevention	Lu Jing
A/Guangdong/036/2014	EPI ISL 192459	8 segments	China	WHO Chinese National Influenza Center	Yang Lei
A/Guangdong/15SF043/2015	EPI ISL 192290	8 segments	China	WHO Chinese National Influenza Center	Yang Lei
A/Guangdong/17SF003/2016	EPI ISL 249309	8 segments	China	WHO Chinese National Influenza Center	Yang Lei
A/Guangdong/17SF006/2017	EPI ISL 249308	8 segments	China	WHO Chinese National Influenza Center	Yang Lei
A/Guangdong/2/2013	EPI ISL 153723	2 segments (4,6)	China	Other Database Import	EpiFluDB
A/Guangdong/24997/2013	EPI ISL 192466	8 segments	China	WHO Chinese National Influenza Center	Yang Lei
A/Guangdong/24999/2013	EPI ISL 192465	8 segments	China	WHO Chinese National Influenza Center	Yang Lei
A/Guangdong/HP001/2017	EPI ISL 256108	8 segments	China	Other Database Import	EpiFluDB
A/Guangdong/SP440/2017	EPI ISL 256109	8 segments	China	Other Database Import	EpiFluDB
A/Guangdong-Guangzhou/XN00457/2014	EPI ISL 192370	8 segments	China	WHO Chinese National Influenza Center	Yang Lei
A/Guangdong-Guangzhou/XN00588/2014	EPI ISL 192358	8 segments	China	WHO Chinese National Influenza Center	Yang Lei
A/Hebei/01/2013	EPI ISL 157293	8 segments	China	WHO Chinese National Influenza Center	Yang Lei
A/Hubei/34007/2015	EPI ISL 192478	8 segments	China	WHO Chinese National Influenza Center	Yang Lei

A/Huizhou/01/2013	EPI ISL 154827	8 segments	China	Other Database Import	EpiFluDB
A/Hunan/02285/2017	EPI ISL 242846	8 segments	China	WHO Chinese National Influenza Center	Yang Lei
A/Hunan/26937/2014	EPI ISL 192382	8 segments	China	WHO Chinese National Influenza Center	Yang Lei
A/jiangsu/03/2013	EPI ISL 141163	8 segments	China	WHO Chinese National Influenza Center	Yang Lei
A/Jiangsu/60456/2016	EPI ISL 242882	8 segments	China	WHO Chinese National Influenza Center	Yang Lei
A/Jiangsu/60457/2016	EPI ISL 242881	8 segments	China	WHO Chinese National Influenza Center	Yang Lei
A/Jiangsu/60466/2016	EPI ISL 242869	8 segments	China	WHO Chinese National Influenza Center	Yang Lei
A/Nanchang/1/2013	EPI ISL 143556	8 segments	China	Other Database Import	EpiFluDB
A/Nanjing/1/2013	EPI ISL 142305	8 segments	China	Other Database Import	EpiFluDB
A/Nanjing/5/2013	EPI ISL 143664	8 segments	China	Jiangsu Provincial Center for Disease Control Prevention	Cui Lunbiao
A/Qingyuan/GIRD1/2017	EPI ISL 249102	8 segments	China	Guangzhou Institute of Respiratory Diseases (GIRD) /<br>State Key Laboratory of Reipiratory Disease (Guangzhou Medical University)	Guan Wenda
A/Quzhou/1/2015	EPI ISL 190691	8 segments	China	Other Database Import	EpiFluDB
A/Shanghai/1/2013	EPI ISL 138737	8 segments	China	WHO Chinese National Influenza Center	Yang Lei
A/Shanghai/Patient6/2013	EPI ISL 142044	8 segments	China	Institute Pasteur of Shanghai CAS /<br>Shanghai public health clinical center	Hao Pei
A/silkie chicken/Dongguan/3606/2013	EPI ISL 175710	8 segments	China	Other Database Import	EpiFluDB
A/silkie chicken/Dongguan/656/2014	EPI ISL 176186	7 segments<br>(1,2,3,4,5,6,7)	China	Other Database Import	EpiFluDB
A/silkie chicken/Jiangxi/9476/2014	EPI ISL 176033	8 segments	China	Other Database Import	EpiFluDB
A/Xinjiang/73030/2014	EPI ISL 192391	8 segments	China	WHO Chinese National Influenza Center	Yang Lei
A/Xinjiang/75802/2014	EPI ISL 192380	8 segments	China	WHO Chinese National Influenza Center	Yang Lei
A/XinjiangBintuan/99117/2014	EPI ISL 172824	8 segments	China	WHO Chinese National Influenza Center	Yang Lei
A/XinjiangBintuan/99118/2014	EPI ISL 172825	8 segments	China	WHO Chinese National Influenza Center	Yang Lei
A/Zhejiang/1/2017	EPI ISL 242856	8 segments	China	WHO Chinese National Influenza Center /<br>Zhejiang Provincial Center for Disease Control and Prevention	Yang Lei
A/Zhejiang/19/2016	EPI ISL 242850	8 segments	China	WHO Chinese National Influenza Center /	Yang Lei

				Zhejiang Provincial Center for Disease Control and Prevention	
A/Zhejiang/22/2013	EPI ISL 148728	8 segments	China	Zhejiang Provincial Center for Disease Control and Prevention	Sun Yi
A/Zhejiang/5/2017	EPI ISL 242848	8 segments	China	WHO Chinese National Influenza Center / Zhejiang Provincial Center for Disease Control and Prevention	Yang Lei
A/Zhejiang/6/2016	EPI ISL 242866	8 segments	China	WHO Chinese National Influenza Center / Zhejiang Provincial Center for Disease Control and Prevention	Yang Lei
A/Zhejiang/6/2017	EPI ISL 242847	8 segments	China	WHO Chinese National Influenza Center / Zhejiang Provincial Center for Disease Control and Prevention	Yang Lei
A/Zhejiang/8/2015	EPI ISL 192497	8 segments	China	WHO Chinese National Influenza Center	Yang Lei
A/Anhui/01881/2014	EPI ISL 192328	8 segments	China	WHO Chinese National Influenza Center	Yang Lei
A/Anhui/01887/2014	EPI ISL 192331	8 segments	China	WHO Chinese National Influenza Center	Yang Lei
A/Anhui/03/2013	EPI ISL 141191	8 segments	China	WHO Chinese National Influenza Center	Yang Lei
A/Anhui/04/2013	EPI ISL 157287	8 segments	China	WHO Chinese National Influenza Center	Yang Lei
A/Anhui/09186/2014	EPI ISL 192405	8 segments	China	WHO Chinese National Influenza Center	Yang Lei
A/Anhui/1-YK RG182/2013	EPI ISL 194449	8 segments	China	Other Database Import	EpiFluDB
A/Anhui/1-YK RG56/2013	EPI ISL 194320	8 segments	China	Other Database Import	EpiFluDB
A/Anhui/33224/2015	EPI ISL 192494	8 segments	China	WHO Chinese National Influenza Center	Yang Lei
A/Anhui/33228/2015	EPI ISL 192492	8 segments	China	WHO Chinese National Influenza Center	Yang Lei
A/Anhui/40094/2015	EPI ISL 192471	8 segments	China	WHO Chinese National Influenza Center	Yang Lei
A/Anhui/40095/2015	EPI ISL 192472	8 segments	China	WHO Chinese National Influenza Center	Yang Lei
A/Anhui/60926/2016	EPI ISL 242900	8 segments	China	WHO Chinese National Influenza Center	Yang Lei
A/Anhui/60927/2016	EPI ISL 242889	8 segments	China	WHO Chinese National Influenza Center	Yang Lei
A/Anhui/60928/2016	EPI ISL 242892	8 segments	China	WHO Chinese National Influenza Center	Yang Lei
A/Anhui/60935/2016	EPI ISL 242898	8 segments	China	WHO Chinese National Influenza Center	Yang Lei
A/Beijing/40610/2015	EPI ISL 192469	8 segments	China	WHO Chinese National Influenza Center	Yang Lei
A/chicken/Anhui-Chuzhou/01/2013	EPI ISL 141161	8 segments	China	WHO Chinese National Influenza Center	Yang Lei
A/chicken/China/028/2014	EPI ISL 208998	8 segments	China	Other Database Import	EpiFluDB
A/chicken/Dongguan/1124/2014	EPI ISL 176359	8 segments	China	Other Database Import	EpiFluDB

A/chicken/Dongguan/1177/2014	EPI ISL 176201	8 segments	China	Other Database Import	EpiFluDB
A/chicken/Dongguan/1307/2014	EPI ISL 176366	8 segments	China	Other Database Import	EpiFluDB
A/chicken/Dongguan/1314/2014	EPI ISL 176367	8 segments	China	Other Database Import	EpiFluDB
A/chicken/Dongguan/1382/2014	EPI ISL 176371	8 segments	China	Other Database Import	EpiFluDB
A/chicken/Dongguan/1421/2014	EPI ISL 176374	8 segments	China	Other Database Import	EpiFluDB
A/chicken/Dongguan/1533/2014	EPI ISL 176409	8 segments	China	Other Database Import	EpiFluDB
A/chicken/Dongguan/1697/2014	EPI ISL 176415	8 segments	China	Other Database Import	EpiFluDB
A/chicken/Dongguan/3141/2013	EPI ISL 175651	8 segments	China	Other Database Import	EpiFluDB
A/chicken/Dongguan/3145/2013	EPI ISL 175652	8 segments	China	Other Database Import	EpiFluDB
A/chicken/Dongguan/4040/2013	EPI ISL 175866	8 segments	China	Other Database Import	EpiFluDB
A/chicken/Dongguan/4064/2013	EPI ISL 175842	8 segments	China	Other Database Import	EpiFluDB
A/chicken/Dongguan/4251/2013	EPI ISL 175734	8 segments	China	Other Database Import	EpiFluDB
A/chicken/Dongguan/864/2014	EPI ISL 176304	8 segments	China	Other Database Import	EpiFluDB
A/chicken/Ganzhou/GZ79/2016	EPI ISL 252837	8 segments	China	Other Database Import	EpiFluDB
A/chicken/Guangdong/3640/2013	EPI ISL 175605	8 segments	China	Other Database Import	EpiFluDB
A/Chicken/Guangdong/CZ145/2015	EPI ISL 176835	8 segments	China	South China Agricultural University	Weixin Jia
A/Chicken/Guangdong/DG120/2015	EPI ISL 176832	8 segments	China	South China Agricultural University	Weixin Jia
A/Chicken/Guangdong/DG478/2014	EPI ISL 176816	8 segments	China	South China Agricultural University	Weixin Jia
A/Chicken/Guangdong/DG478/2014	EPI ISL 176816	8 segments	China	South China Agricultural University	Weixin Jia(2)
A/Chicken/Guangdong/DG479/2014	EPI ISL 176817	8 segments	China	South China Agricultural University	Weixin Jia
A/Chicken/Guangdong/DG592/2014	EPI ISL 176819	8 segments	China	South China Agricultural University	Weixin Jia
A/Chicken/Guangdong/DG593/2014	EPI ISL 176820	8 segments	China	South China Agricultural University	Weixin Jia
A/chicken/Guangdong/G1/2013	EPI ISL 175606	8 segments	China	Other Database Import	EpiFluDB
A/chicken/Guangdong/G3/2013	EPI ISL 175608	8 segments	China	Other Database Import	EpiFluDB
A/Chicken/Guangdong/GZ068/2015	EPI ISL 176834	8 segments	China	South China Agricultural University	Weixin Jia
A/Chicken/Guangdong/HZ098/2015	EPI ISL 176824	8 segments	China	South China Agricultural University	Weixin Jia
A/chicken/Guangdong/SD1/2013	EPI ISL 175609	8 segments	China	Other Database Import	EpiFluDB
A/chicken/Guangdong/SD641/2013	EPI ISL 142934	8 segments	China	Harbin Veterinary Research Institute (CAAS)	Kong Huihui

A/Chicken/Guangdong/SW153/2015	EPI ISL 176836	8 segments	China	South China Agricultural University	Weixin Jia
A/Chicken/Guangdong/SW154/2015	EPI ISL 176837	8 segments	China	South China Agricultural University	Weixin Jia
A/chicken/Guangzhou/1/2013	EPI ISL 157153	1 segments (4)	China	Other Database Import	EpiFluDB
A/chicken/Hangzhou/174/2013	EPI ISL 194993	8 segments	China	Other Database Import	EpiFluDB
A/chicken/Henan/102/2013	EPI ISL 206699	8 segments	China	Other Database Import	EpiFluDB
A/chicken/Henan/115/2013	EPI ISL 206706	8 segments	China	Other Database Import	EpiFluDB
A/chicken/Henan/120/2013	EPI ISL 206708	8 segments	China	Other Database Import	EpiFluDB
A/chicken/Henan/141/2013	EPI ISL 206709	8 segments	China	Other Database Import	EpiFluDB
A/chicken/Huzhou/3791/2013	EPI ISL 175738	8 segments	China	Other Database Import	EpiFluDB
A/chicken/Huzhou/3802/2013	EPI ISL 175739	8 segments	China	Other Database Import	EpiFluDB
A/chicken/Huzhou/4067/2013	EPI ISL 175769	8 segments	China	Other Database Import	EpiFluDB
A/chicken/Huzhou/4074/2013	EPI ISL 175772	8 segments	China	Other Database Import	EpiFluDB
A/chicken/Huzhou/4076/2013	EPI ISL 175773	8 segments	China	Other Database Import	EpiFluDB
A/chicken/Jiangsu/1021/2013	EPI ISL 153841	8 segments	China	Other Database Import	EpiFluDB
A/chicken/Jiangsu/SC035/2013	EPI ISL 142932	8 segments	China	Harbin Veterinary Research Institute (CAAS)	Kong Huihui
A/chicken/Jiangsu/SC537/2013	EPI ISL 142930	8 segments	China	Harbin Veterinary Research Institute (CAAS)	Kong Huihui
A/chicken/Jiangxi/10885/2014	EPI ISL 176047	8 segments	China	Other Database Import	EpiFluDB
A/chicken/Jiangxi/10896/2014	EPI ISL 176050	8 segments	China	Other Database Import	EpiFluDB
A/chicken/Jiangxi/10964/2014	EPI ISL 176131	8 segments	China	Other Database Import	EpiFluDB
A/chicken/Jiangxi/12200/2014	EPI ISL 179904	8 segments	China	Other Database Import	EpiFluDB
A/chicken/Jiangxi/12208/2014	EPI ISL 179960	8 segments	China	Other Database Import	EpiFluDB
A/chicken/Jiangxi/12219/2014	EPI ISL 179961	8 segments	China	Other Database Import	EpiFluDB
A/chicken/Jiangxi/12245/2014	EPI ISL 179856	8 segments	China	Other Database Import	EpiFluDB
A/chicken/Jiangxi/12249/2014	EPI ISL 179854	8 segments	China	Other Database Import	EpiFluDB
A/chicken/Jiangxi/12251/2014	EPI ISL 179968	8 segments	China	Other Database Import	EpiFluDB
A/chicken/Jiangxi/12261/2014	EPI ISL 179859	8 segments	China	Other Database Import	EpiFluDB
A/chicken/Jiangxi/12264/2014	EPI ISL 179971	8 segments	China	Other Database Import	EpiFluDB
A/chicken/Jiangxi/12544/2013	EPI ISL 175827	8 segments	China	Other Database Import	EpiFluDB

A/chicken/Jiangxi/12564/2013	EPI ISL 179799	8 segments	China	Other Database Import	EpiFluDB
A/chicken/Jiangxi/13502/2014	EPI ISL 179912	8 segments	China	Other Database Import	EpiFluDB
A/chicken/Jiangxi/13507/2014	EPI ISL 179979	8 segments	China	Other Database Import	EpiFluDB
A/chicken/Jiangxi/13510/2014	EPI ISL 179917	8 segments	China	Other Database Import	EpiFluDB
A/chicken/Jiangxi/13524/2014	EPI ISL 179984	8 segments	China	Other Database Import	EpiFluDB
A/chicken/Jiangxi/13543/2014	EPI ISL 180020	8 segments	China	Other Database Import	EpiFluDB
A/chicken/Jiangxi/13548/2014	EPI ISL 180022	8 segments	China	Other Database Import	EpiFluDB
A/chicken/Jiangxi/13553/2014	EPI ISL 179920	8 segments	China	Other Database Import	EpiFluDB
A/chicken/Jiangxi/14033/2014	EPI ISL 180028	8 segments	China	Other Database Import	EpiFluDB
A/chicken/Jiangxi/14517/2014	EPI ISL 180036	8 segments	China	Other Database Import	EpiFluDB
A/chicken/Jiangxi/14518/2014	EPI ISL 180037	8 segments	China	Other Database Import	EpiFluDB
A/chicken/Jiangxi/15524/2014	EPI ISL 180085	8 segments	China	Other Database Import	EpiFluDB
A/chicken/Jiangxi/18008/2014	EPI ISL 180100	8 segments	China	Other Database Import	EpiFluDB
A/chicken/Jiangxi/18482/2014	EPI ISL 180141	8 segments	China	Other Database Import	EpiFluDB
A/chicken/Jiangxi/18487/2014	EPI ISL 180145	8 segments	China	Other Database Import	EpiFluDB
A/chicken/Jiangxi/18513/2014	EPI ISL 180148	8 segments	China	Other Database Import	EpiFluDB
A/chicken/Jiangxi/9534/2014	EPI ISL 176035	8 segments	China	Other Database Import	EpiFluDB
A/chicken/Jiangxi/SD001/2013	EPI ISL 142929	8 segments	China	Harbin Veterinary Research Institute (CAAS)	Kong Huihui
A/chicken/Jiaxing/4490/2013	EPI ISL 175784	8 segments	China	Other Database Import	EpiFluDB
A/chicken/Longquan/LQ78/2016	EPI ISL 252829	8 segments	China	Other Database Import	EpiFluDB
A/Chicken/Nanjing/761/2013	EPI ISL 159062	8 segments	China	Beijing Institute of Microbiology and Epidemiology	Fan Hang
A/chicken/Quzhou/2/2015	EPI ISL 190693	8 segments	China	Other Database Import	EpiFluDB
A/chicken/Shantou/4325/2014	EPI ISL 180093	8 segments	China	Other Database Import	EpiFluDB
A/chicken/Shantou/4824/2014	EPI ISL 180094	8 segments	China	Other Database Import	EpiFluDB
A/chicken/Shaoxing/2417/2013	EPI ISL 175736	8 segments	China	Other Database Import	EpiFluDB
A/chicken/Shaoxing/5087/2013	EPI ISL 175788	8 segments	China	Other Database Import	EpiFluDB
A/chicken/Shaoxing/5201/2013	EPI ISL 175817	8 segments	China	Other Database Import	EpiFluDB
A/chicken/Shaoxing/5227/2013	EPI ISL 175819	8 segments	China	Other Database Import	EpiFluDB

A/chicken/Shaoxing/5479/2013	EPI ISL 175822	8 segments	China	Other Database Import	EpiFluDB
A/chicken/Shenzhen/2293/2013	EPI ISL 175647	8 segments	China	Other Database Import	EpiFluDB
A/chicken/Shenzhen/3733/2013	EPI ISL 175711	8 segments	China	Other Database Import	EpiFluDB
A/chicken/Shenzhen/3734/2013	EPI ISL 175712	8 segments	China	Other Database Import	EpiFluDB
A/chicken/Taizhou/TZJF02/2015	EPI ISL 205125	8 segments	China	Other Database Import	EpiFluDB
A/chicken/Wenzhou/HATSLG01/2015	EPI ISL 205127	8 segments	China	Other Database Import	EpiFluDB
A/chicken/Wenzhou/RAQL01/2015	EPI ISL 205122	8 segments	China	Other Database Import	EpiFluDB
A/chicken/Wenzhou/RAQL18/2015	EPI ISL 205123	8 segments	China	Other Database Import	EpiFluDB
A/chicken/Wenzhou/WZTSLG02/2015	EPI ISL 205128	8 segments	China	Other Database Import	EpiFluDB
A/chicken/Wuxi/0405005/2013	EPI ISL 200115	8 segments	China	Other Database Import	EpiFluDB
A/chicken/Zhangzhou/8585/2014	EPI ISL 180156	8 segments	China	Other Database Import	EpiFluDB
A/chicken/Zhejiang/C483/2013	EPI ISL 162867	8 segments	China	Other Database Import	EpiFluDB
A/chicken/Zhejiang/DTID-ZJU01/2013	EPI ISL 139501	8 segments	China	Other Database Import	EpiFluDB
A/chicken/Zhejiang/SD007/2013	EPI ISL 142919	8 segments	China	Harbin Veterinary Research Institute (CAAS)	Kong Huihui
A/Duck/Guangdong/DG103/2015	EPI ISL 176828	8 segments	China	South China Agricultural University	Weixin Jia
A/Duck/Guangdong/DG527/2014	EPI ISL 176818	8 segments	China	South China Agricultural University	Weixin Jia
A/duck/Wenzhou/RAQL10/2015	EPI ISL 205124	8 segments	China	Other Database Import	EpiFluDB
A/duck/Wenzhou/YJYF24/2015	EPI ISL 205126	8 segments	China	Other Database Import	EpiFluDB
A/Fujian/02/2013	EPI ISL 157288	8 segments	China	WHO Chinese National Influenza Center	Yang Lei
A/Fujian/02151/2017	EPI ISL 242842	8 segments	China	WHO Chinese National Influenza Center / Fujian Provincial Center for Disease Control and Prevention	Yang Lei
A/Fujian/02152/2017	EPI ISL 242843	8 segments	China	WHO Chinese National Influenza Center / Fujian Provincial Center for Disease Control and Prevention	Yang Lei
A/Fujian/03/2013	EPI ISL 157289	8 segments	China	WHO Chinese National Influenza Center	Yang Lei
A/Fujian/04/2013	EPI ISL 157290	8 segments	China	WHO Chinese National Influenza Center	Yang Lei
A/Fujian/05/2013	EPI ISL 157292	8 segments	China	WHO Chinese National Influenza Center	Yang Lei
A/Fujian/1/2015	EPI ISL 192335	8 segments	China	WHO Chinese National Influenza Center	Yang Lei
A/Fujian/1/2016	EPI ISL 233628	8 segments	China	Fujian Center for Disease Control and Prevention	Jianfeng Xie

A/Fujian/17/2014	EPI ISL 192336	8 segments	China	WHO Chinese National Influenza Center	Yang Lei
A/Fujian/17/2015	EPI ISL 192289	8 segments	China	WHO Chinese National Influenza Center	Yang Lei
A/Fujian/22/2014	EPI ISL 192322	8 segments	China	WHO Chinese National Influenza Center	Yang Lei
A/Fujian/3/2016	EPI ISL 233629	8 segments	China	Fujian Center for Disease Control and Prevention	Jianfeng Xie
A/Fujian/54840/2016	EPI ISL 242901	8 segments	China	WHO Chinese National Influenza Center	Yang Lei
A/Fujian/56600/2016	EPI ISL 242872	8 segments	China	WHO Chinese National Influenza Center /Fujian Provincial Center for Disease Control and Prevention	Yang Lei
A/Fujian/9/2015	EPI ISL 192311	8 segments	China	WHO Chinese National Influenza Center	Yang Lei
A/Guangdong/02496/2014	EPI ISL 172823	8 segments	China	WHO Chinese National Influenza Center	Yang Lei
A/Guangdong/02497/2014	EPI ISL 192324	8 segments	China	WHO Chinese National Influenza Center	Yang Lei
A/Guangdong/02620/2014	EPI ISL 192464	8 segments	China	WHO Chinese National Influenza Center	Yang Lei
A/Guangdong/035/2014	EPI ISL 192460	8 segments	China	WHO Chinese National Influenza Center	Yang Lei
A/Guangdong/15SF002/2015	EPI ISL 192302	8 segments	China	WHO Chinese National Influenza Center	Yang Lei
A/Guangdong/15SF020/2015	EPI ISL 192300	8 segments	China	WHO Chinese National Influenza Center	Yang Lei
A/Guangdong/15SF053/2015	EPI ISL 192284	8 segments	China	WHO Chinese National Influenza Center	Yang Lei
A/Guangdong/15SF080/2015	EPI ISL 192276	8 segments	China	WHO Chinese National Influenza Center	Yang Lei
A/Guangdong/15SF081/2015	EPI ISL 192282	8 segments	China	WHO Chinese National Influenza Center	Yang Lei
A/Guangdong/15SF082/2015	EPI ISL 192275	8 segments	China	WHO Chinese National Influenza Center	Yang Lei
A/Guangdong/17SF003/2016	EPI ISL 249309	8 segments	China	WHO Chinese National Influenza Center	Yang Lei
A/Guangdong/17SF006/2017	EPI ISL 249308	8 segments	China	WHO Chinese National Influenza Center	Yang Lei
A/Guangdong/24997/2013	EPI ISL 192466	8 segments	China	WHO Chinese National Influenza Center	Yang Lei
A/Guangdong/24999/2013	EPI ISL 192465	8 segments	China	WHO Chinese National Influenza Center	Yang Lei
A/Guangdong/60061/2016	EPI ISL 242888	8 segments	China	WHO Chinese National Influenza Center / Guangdong Provincial Center for Disease Control and Prevention	Yang Lei
A/Guangdong/60923/2016	EPI ISL 242870	8 segments	China	WHO Chinese National Influenza Center / Guangdong Provincial Center for Disease Control and Prevention	Yang Lei
A/Guangdong/DG-02/2013	EPI ISL 159070	8 segments	China	Guangdong Provincial Center for Disease Control and Prevention	Lu Jing
A/Guangdong/DG-03/2013	EPI ISL 159071	8 segments	China	Guangdong Provincial Center for Disease Control and Prevention	Lu Jing

A/Guangdong/YJ-04/2013	EPI ISL 159072	8 segments	China	Guangdong Provincial Center for Disease Control and Prevention	Lu Jing
A/Guangdong/YJ-05/2013	EPI ISL 159073	8 segments	China	Guangdong Provincial Center for Disease Control and Prevention	Lu Jing
A/Hangzhou/254/2013	EPI ISL 141775	8 segments	China	Hangzhou Center for Disease Control and Prevention	Li Jun
A/Hebei/01/2013	EPI ISL 157293	8 segments	China	WHO Chinese National Influenza Center	Yang Lei
A/Hubei/34007/2015	EPI ISL 192478	8 segments	China	WHO Chinese National Influenza Center	Yang Lei
A/Huizhou/01/2013	EPI ISL 154827	8 segments	China	Other Database Import	EpiFluDB
A/Hunan/02285/2017	EPI ISL 242846	8 segments	China	WHO Chinese National Influenza Center	Yang Lei
A/Hunan/02286/2017	EPI ISL 242844	8 segments	China	WHO Chinese National Influenza Center	Yang Lei
A/Hunan/02287/2017	EPI ISL 242845	8 segments	China	WHO Chinese National Influenza Center	Yang Lei
A/Hunan/08963/2014	EPI ISL 192438	8 segments	China	WHO Chinese National Influenza Center	Yang Lei
A/Hunan/09193/2014	EPI ISL 192424	8 segments	China	WHO Chinese National Influenza Center	Yang Lei
A/Hunan/19762/2015	EPI ISL 192286	8 segments	China	WHO Chinese National Influenza Center	Yang Lei
A/Hunan/26938/2014	EPI ISL 192383	8 segments	China	WHO Chinese National Influenza Center	Yang Lei
A/Jiangsu/06307/2014	EPI ISL 172829	8 segments	China	WHO Chinese National Influenza Center	Yang Lei
A/Jiangsu/22184/2015	EPI ISL 192274	8 segments	China	WHO Chinese National Influenza Center	Yang Lei
A/Jiangsu/60450/2016	EPI ISL 242886	8 segments	China	WHO Chinese National Influenza Center	Yang Lei
A/Jiangsu/60451/2016	EPI ISL 242885	8 segments	China	WHO Chinese National Influenza Center	Yang Lei
A/Jiangsu/60456/2016	EPI ISL 242882	8 segments	China	WHO Chinese National Influenza Center	Yang Lei
A/Jiangsu/60457/2016	EPI ISL 242881	8 segments	China	WHO Chinese National Influenza Center	Yang Lei
A/Jiangsu/60458/2016	EPI ISL 242880	8 segments	China	WHO Chinese National Influenza Center	Yang Lei
A/Jiangsu/60459/2016	EPI ISL 242879	8 segments	China	WHO Chinese National Influenza Center	Yang Lei
A/Jiangsu/60461/2016	EPI ISL 242877	8 segments	China	WHO Chinese National Influenza Center	Yang Lei
A/Jiangsu/60462/2016	EPI ISL 242876	8 segments	China	WHO Chinese National Influenza Center	Yang Lei
A/Jiangsu/60465/2016	EPI ISL 242874	8 segments	China	WHO Chinese National Influenza Center	Yang Lei
A/Jiangsu/60466/2016	EPI ISL 242869	8 segments	China	WHO Chinese National Influenza Center	Yang Lei
A/Jiangsu/60467/2016	EPI ISL 242873	8 segments	China	WHO Chinese National Influenza Center	Yang Lei
A/Jiangsu/Wuxi04/2013	EPI ISL 220945	8 segments	China	Beijing Institute of Microbiology and Epidemiology	Ma Maijuan
A/Minhang/S01/2013	EPI ISL 252548	8 segments	China	Other Database Import	EpiFluDB

A/Nanjing/6/2013	EPI ISL 153011	8 segments	China	Other Database Import	EpiFluDB
A/Nanjing/7/2013	EPI ISL 153013	8 segments	China	Other Database Import	EpiFluDB
A/pigeon/Shanghai/S1421/2013	EPI ISL 142903	8 segments	China	Harbin Veterinary Research Institute (CAAS)	Kong Huihui
A/Qingyuan/GIRD1/2017	EPI ISL 249102	8 segments	China	Guangzhou Institute of Respiratory Diseases (GIRD) /State Key Laboratory of Reipiratory Disease (Guangzhou Medical University)	Guan Wenda
A/Quzhou/1/2015	EPI ISL 190691	8 segments	China	Other Database Import	EpiFluDB
A/Quzhou/2/2015	EPI ISL 190692	8 segments	China	Other Database Import	EpiFluDB
A/Shandong/01/2014	EPI ISL 192377	8 segments	China	WHO Chinese National Influenza Center	Yang Lei
A/Shandong-Taian/01/2015	EPI ISL 192499	8 segments	China	WHO Chinese National Influenza Center	Yang Lei
A/Shanghai/01/2014	EPI ISL 162470	8 segments	China	Other Database Import	EpiFluDB
A/Shanghai/02/2013	EPI ISL 141437	8 segments	China	Other Database Import	EpiFluDB
A/Shanghai/1/2013	EPI ISL 138737	8 segments	China	WHO Chinese National Influenza Center	Yang Lei
A/Shanghai/3/2013	EPI ISL 139649	8 segments	China	WHO Chinese National Influenza Center	Yang Lei
A/Shanghai/4664T/2013	EPI ISL 140924	8 segments	China	Other Database Import	EpiFluDB
A/Shanghai/ION/2013	EPI ISL 177875	8 segments	China	Other Database Import	EpiFluDB
A/Shanghai/MH01/2013	EPI ISL 166490	8 segments	China	Other Database Import	EpiFluDB
A/Shantou/1002/2014	EPI ISL 162619	8 segments	China	Shantou University Medical College	Farooqui Amber
A/Shenzhen/SP126/2014	EPI ISL 179907	8 segments	China	Other Database Import	EpiFluDB
A/Shenzhen/SP139/2014	EPI ISL 179906	8 segments	China	Other Database Import	EpiFluDB
A/silkie chicken/Dongguan/1641/2014	EPI ISL 176411	8 segments	China	Other Database Import	EpiFluDB
A/silkie chicken/Dongguan/3049/2013	EPI ISL 175649	8 segments	China	Other Database Import	EpiFluDB
A/silkie chicken/Dongguan/3605/2013	EPI ISL 175709	8 segments	China	Other Database Import	EpiFluDB
A/silkie chicken/Dongguan/3606/2013	EPI ISL 175710	8 segments	China	Other Database Import	EpiFluDB
A/silkie chicken/Huzhou/4213/2013	EPI ISL 175781	8 segments	China	Other Database Import	EpiFluDB
A/silkie chicken/Shaoxing/5130/2013	EPI ISL 175789	8 segments	China	Other Database Import	EpiFluDB
A/silkie chicken/Shaoxing/5235/2013	EPI ISL 175820	8 segments	China	Other Database Import	EpiFluDB
A/silkie chicken/Shenzhen/3781/2013	EPI ISL 175714	8 segments	China	Other Database Import	EpiFluDB
A/silkie chicken/Shenzhen/3782/2013	EPI ISL 175715	8 segments	China	Other Database Import	EpiFluDB

A/Suzhou/3/2013	EPI ISL 153010	8 segments	China	Other Database Import	EpiFluDB
A/Suzhou/5/2013	EPI ISL 153012	8 segments	China	Other Database Import	EpiFluDB
A/tree sparrow/Shanghai/01/2013	EPI ISL 156527	8 segments	China	Other Database Import	EpiFluDB
A/wild pigeon/Jiangsu/SD001/2013	EPI ISL 142901	8 segments	China	Harbin Veterinary Research Institute (CAAS)	Kong Huihui
A/Wuxi/2/2013	EPI ISL 145652	8 segments	China	Other Database Import	EpiFluDB
A/Xinjiang/05916/2014	EPI ISL 172818	8 segments	China	WHO Chinese National Influenza Center	Yang Lei
A/Xinjiang/73030/2014	EPI ISL 192391	8 segments	China	WHO Chinese National Influenza Center	Yang Lei
A/Xinjiang/75802/2014	EPI ISL 192380	8 segments	China	WHO Chinese National Influenza Center	Yang Lei
A/Xinjiang/98692/2014	EPI ISL 172826	8 segments	China	WHO Chinese National Influenza Center	Yang Lei
A/XinjiangBintuan/99117/2014	EPI ISL 172824	8 segments	China	WHO Chinese National Influenza Center	Yang Lei
A/Zhejiang/1/2013	EPI ISL 139652	8 segments	China	Zhejiang Provincial Center for Disease Control and Prevention	Sun Yi
A/Zhejiang/1/2017	EPI ISL 242856	8 segments	China	WHO Chinese National Influenza Center / Zhejiang Provincial Center for Disease Control and Prevention	Yang Lei
A/Zhejiang/10/2016	EPI ISL 242863	8 segments	China	WHO Chinese National Influenza Center / Zhejiang Provincial Center for Disease Control and Prevention	Yang Lei
A/Zhejiang/11/2015	EPI ISL 192503	8 segments	China	WHO Chinese National Influenza Center	Yang Lei
A/Zhejiang/12/2016	EPI ISL 242861	8 segments	China	WHO Chinese National Influenza Center / Zhejiang Provincial Center for Disease Control and Prevention	Yang Lei
A/Zhejiang/13/2016	EPI ISL 242860	8 segments	China	WHO Chinese National Influenza Center / Zhejiang Provincial Center for Disease Control and Prevention	Yang Lei
A/Zhejiang/15/2016	EPI ISL 242858	8 segments	China	WHO Chinese National Influenza Center / Zhejiang Provincial Center for Disease Control and Prevention	Yang Lei
A/Zhejiang/17/2014	EPI ISL 169131	8 segments	China	Zhejiang Provincial Center for Disease Control and Prevention	Sun Yi
A/Zhejiang/17/2016	EPI ISL 242853	8 segments	China	WHO Chinese National Influenza Center / Zhejiang Provincial Center for Disease Control and Prevention	Yang Lei
A/Zhejiang/18/2016	EPI ISL 242852	8 segments	China	WHO Chinese National Influenza Center / Zhejiang Provincial Center for Disease Control and Prevention	Yang Lei
A/Zhejiang/2/2017	EPI ISL 242855	8 segments	China	WHO Chinese National Influenza Center /	Yang Lei

				Zhejiang Provincial Center for Disease Control and Prevention	
A/Zhejiang/20/2016	EPI ISL 242849	8 segments	China	WHO Chinese National Influenza Center / Zhejiang Provincial Center for Disease Control and Prevention	Yang Lei
A/Zhejiang/3/2017	EPI ISL 242867	8 segments	China	WHO Chinese National Influenza Center / Zhejiang Provincial Center for Disease Control and Prevention	Yang Lei
A/Zhejiang/32/2013	EPI ISL 148299	3 segments (4,7,8)	China	Other Database Import	EpiFluDB
A/Zhejiang/36/2014	EPI ISL 192292	8 segments	China	WHO Chinese National Influenza Center	Yang Lei
A/Zhejiang/4/2017	EPI ISL 242851	8 segments	China	WHO Chinese National Influenza Center /Zhejiang Provincial Center for Disease Control and Prevention	Yang Lei
A/Zhejiang/5/2017	EPI ISL 242848	8 segments	China	WHO Chinese National Influenza Center / Zhejiang Provincial Center for Disease Control and Prevention	Yang Lei
A/Zhejiang/6/2016	EPI ISL 242866	8 segments	China	WHO Chinese National Influenza Center / Zhejiang Provincial Center for Disease Control and Prevention	Yang Lei
A/Zhejiang/6/2017	EPI ISL 242847	8 segments	China	WHO Chinese National Influenza Center / Zhejiang Provincial Center for Disease Control and Prevention	Yang Lei
A/Zhejiang/7/2015	EPI ISL 192498	8 segments	China	WHO Chinese National Influenza Center	Yang Lei
A/Zhejiang/7/2016	EPI ISL 242854	8 segments	China	WHO Chinese National Influenza Center / Zhejiang Provincial Center for Disease Control and Prevention	Yang Lei
A/Zhejiang/8/2015	EPI ISL 192497	8 segments	China	WHO Chinese National Influenza Center	Yang Lei

Supplementary Figure S1. S1 shows phylogenetic trees of PB2 and PB1 genes of H7N9 viruses. Solid red circles indicate CK/J1 and CK/J2 found in this study. Solid green triangles indicate avian source H7N9 strains. Solid blue squares indicate human source H7N9 strains. Strains detected in the Yangtze River Delta region are indicated by red lines, whereas those detected in the Pearl River Delta region are indicated by blue lines. Strains found in regions other than the above two areas were considered to be from other regions and are indicated by green lines. Yellow shadows show novel human HP strains found in 2017 in Guangdong; the blue shadow shows the two chicken strains found in this study. All sequences involved in phylogenetic trees were aligned using Clustal W and built using the neighbor-joining method (1000 replicates) in MEGA 7.0. Abbreviations: YRD (Yangtze River Delta), PRD (Pearl River Delta), OR (other regions), W3 (the 3rd wave epidemic), W4 (the 4th wave epidemic).

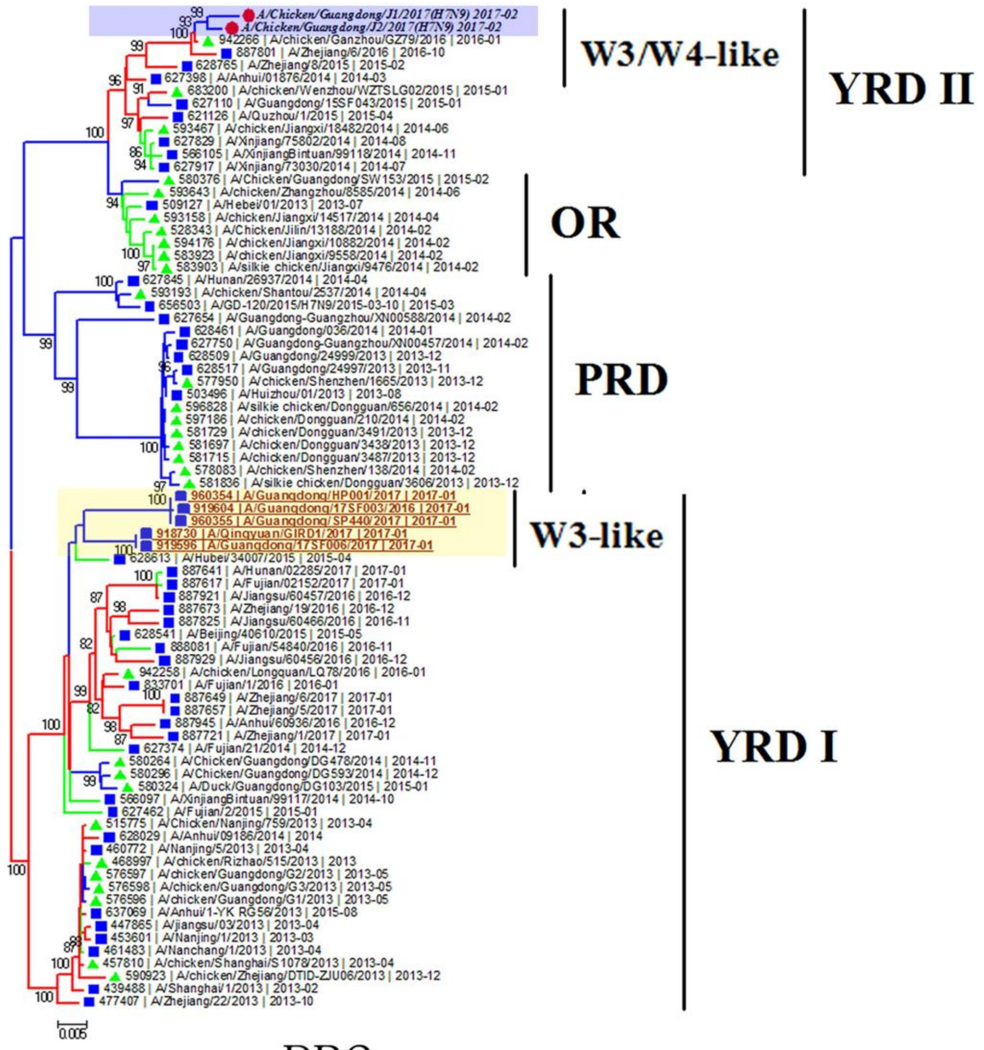
W3/W4-like
YRD II
OR
PRD
W3-like
YRD I
0.005

PB2

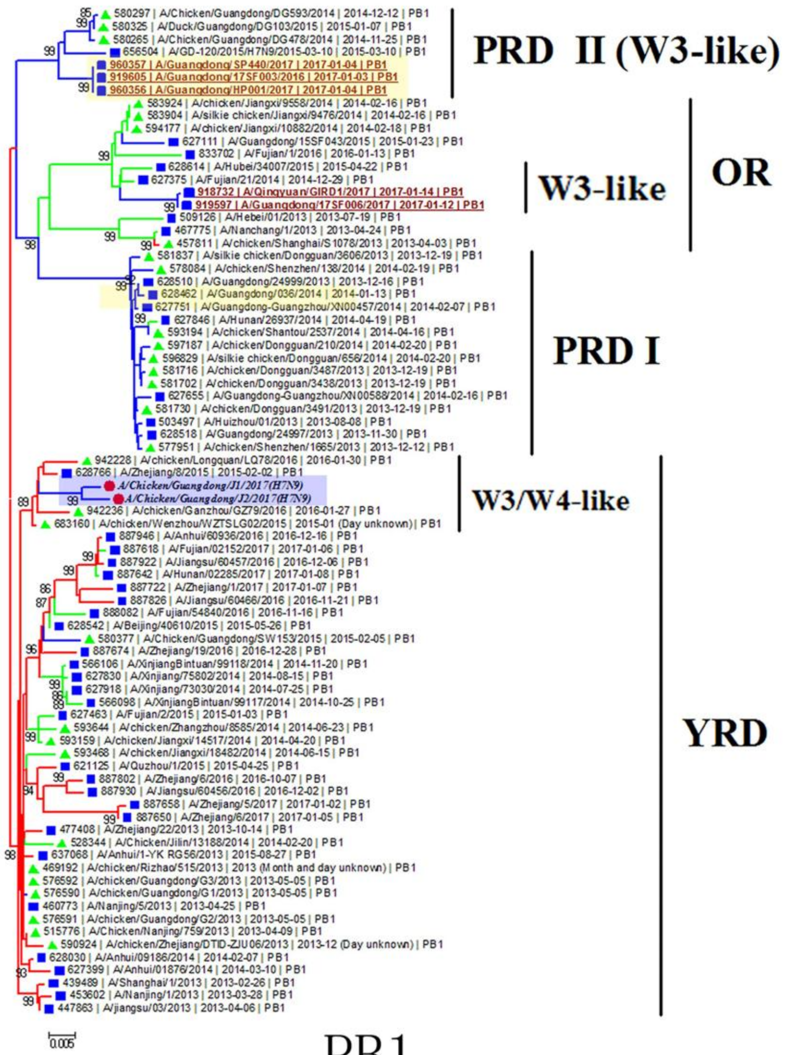
PRD II (W3-like)
W3-like
OR
PRD I
W3/W4-like
YRD
0.005

PB1

Supplementary Figure S2. S2 shows phylogenetic trees of PA and HA genes of H7N9 viruses. Solid red circles indicate CK/J1 and CK/J2 found in this study. Solid green triangles indicate avian H7N9 strains. Solid blue squares indicate human H7N9 strains. Strains detected in the Yangtze River Delta region are indicated by red lines, whereas those detected in the Pearl River Delta region are indicated by blue lines. Strains found in regions other than the above two areas were considered to be from other regions and are indicated by green lines. Yellow shadows show novel human HP strains found in 2017 in Guangdong; the blue shadow shows the two chicken strains found in this study. All sequences involved in phylogenetic trees were aligned using Clustal W and built using the neighbor-joining method (1000 replicates) in MEGA 7.0. Abbreviations: YRD (Yangtze River Delta), PRD (Pearl River Delta), OR (other regions), W3 (the 3rd wave epidemic), W4 (the 4th wave epidemic).

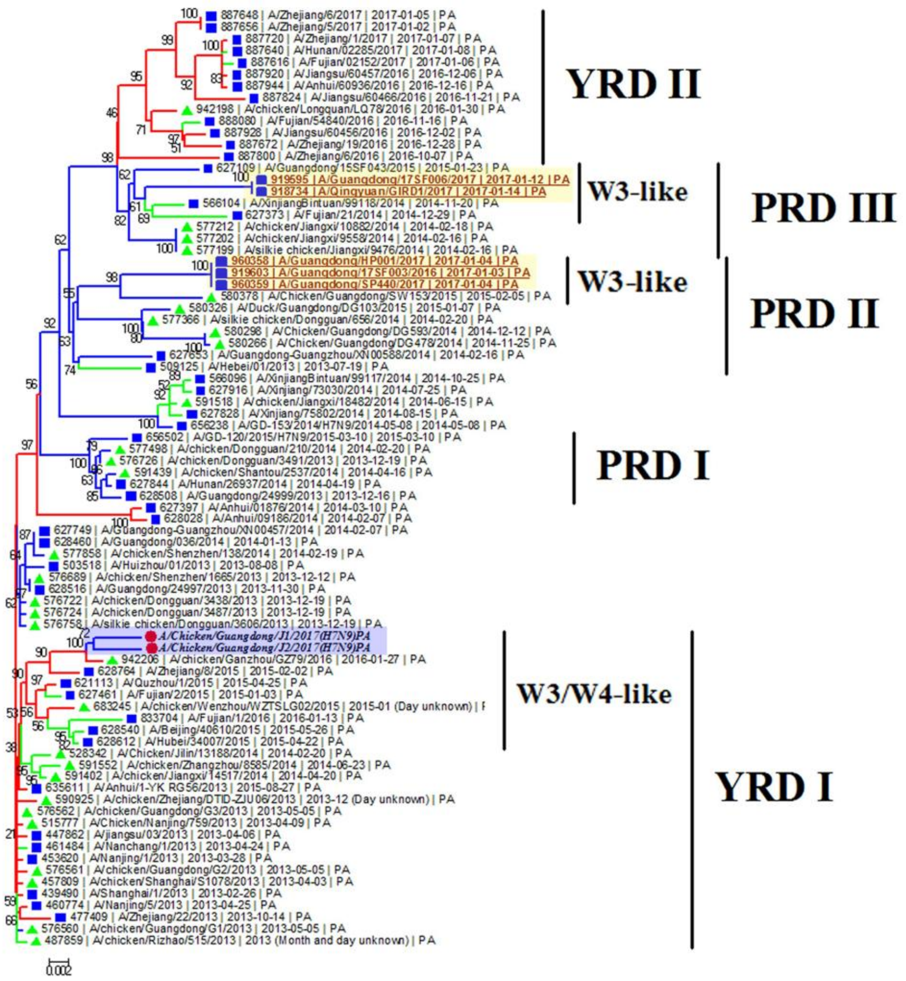
YRD II
W3-like
PRD III
W3-like
PRD II
PRD I
W3/W4-like
YRD I
0.002

PA

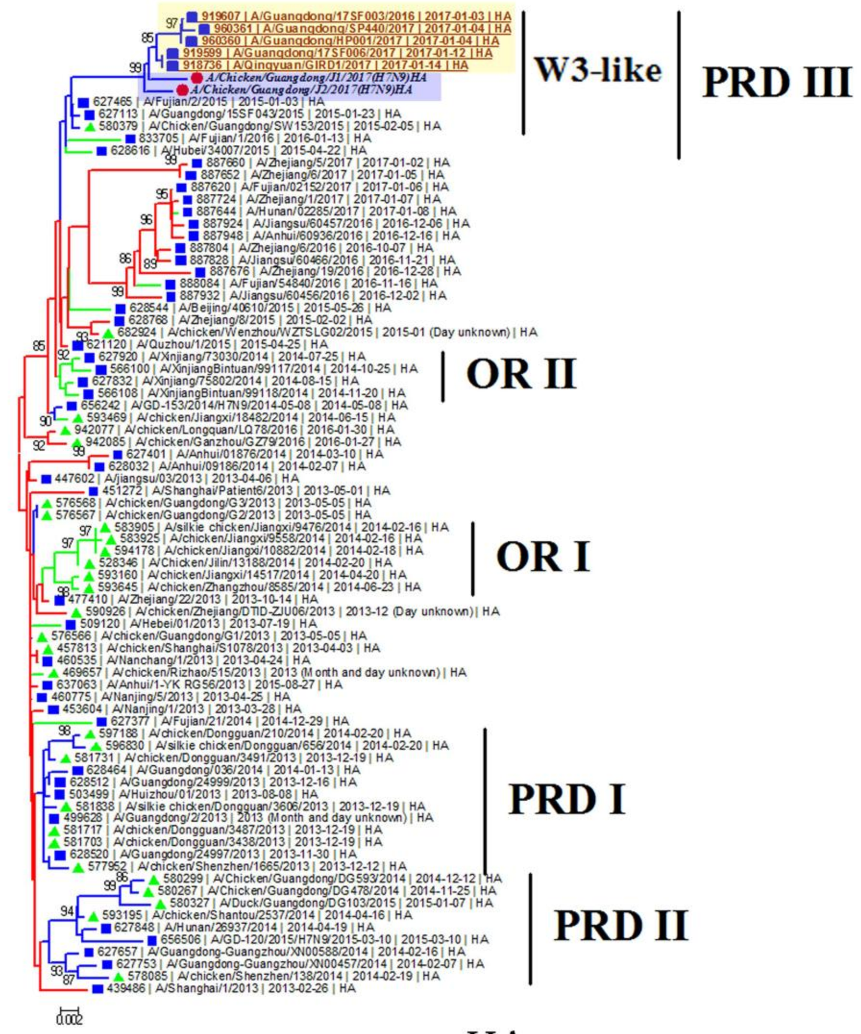
W3-like
PRD III
OR II
OR I
PRD I
PRD II
0.002

HA

Supplementary Figure S3. S3 shows phylogenetic trees of NP and NA genes of H7N9 viruses. Solid red circles indicate CK/J1 and CK/J2 found in this study. Solid green triangles indicate avian H7N9 strains. Solid blue squares indicate human H7N9 strains. Strains detected in the Yangtze River Delta region are indicated by red lines, whereas those detected in the Pearl River Delta region are indicated by blue lines. Strains found in regions other than the above two areas were considered to be from other regions and are indicated by green lines. Yellow shadows show novel human HP strains found in 2017 in Guangdong; the blue shadow shows the two chicken strains found in this study. All sequences involved in phylogenetic trees were aligned using Clustal W and built using the neighbor-joining method (1000 replicates) in MEGA 7.0. Abbreviations: YRD (Yangtze River Delta), PRD (Pearl River Delta), OR (other regions), W3 (the 3rd wave epidemic), W5 (the 5th wave epidemic).

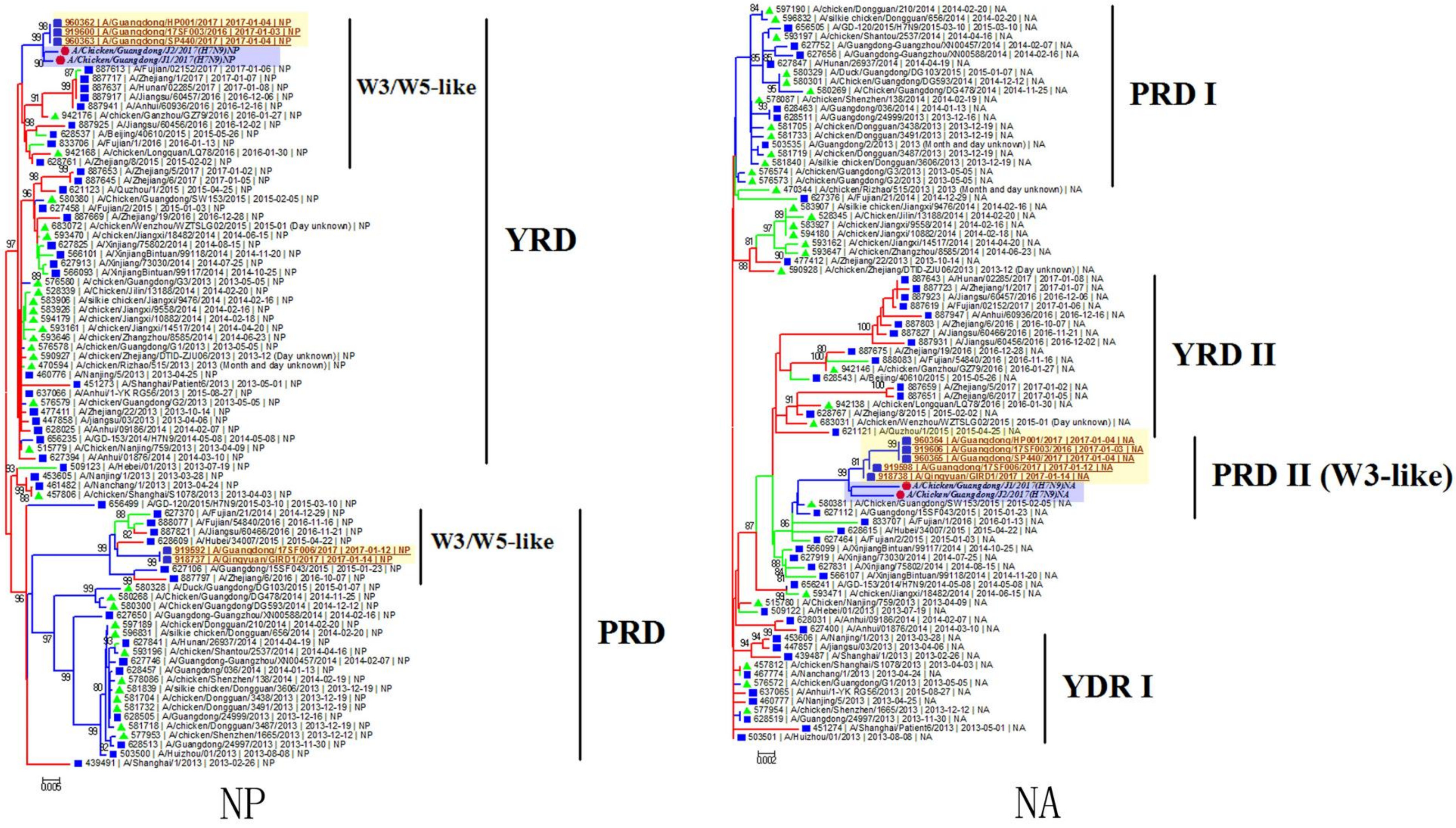
W3/W5-like
YRD
W3/W5-like
PRD
NP
PRD I
YRD II
PRD II (W3-like)
YDR I
NA

Supplementary Figure S4. S4 shows phylogenetic trees of M and NS genes of H7N9 viruses. Solid red circles indicate CK/J1 and CK/J2 found in this study. Solid green triangles indicate avian H7N9 strains. Solid blue squares indicate human H7N9 strains. Strains detected in the Yangtze River Delta region are indicated by red lines, whereas those detected in the Pearl River Delta region are indicated by blue lines. Strains found in regions other than the above two areas were considered to be from other regions and are indicated by green lines. Yellow shadows show novel human HP strains found in 2017 in Guangdong; the blue shadow shows the two chicken strains found in this study. All sequences involved in phylogenetic trees were aligned using Clustal W and built using the neighbor-joining method (1000 replicates) in MEGA 7.0. Abbreviations: YRD (Yangtze River Delta), PRD (Pearl River Delta), OR (other regions), W1, W2, W3, W4 and W5 (the 1st to 5th wave epidemic).

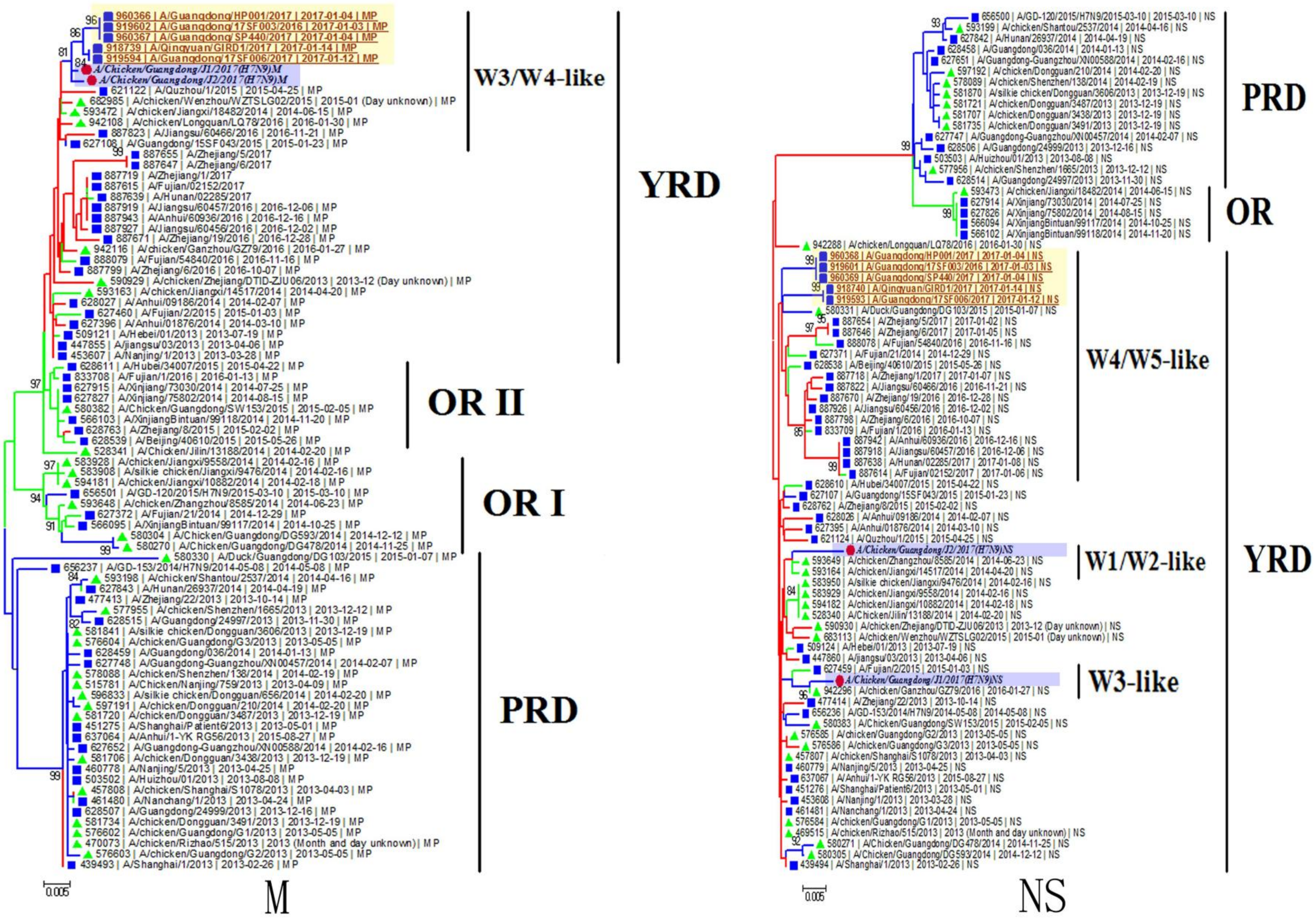
W3/W4-like
YRD
OR II
OR I
PRD
0.005
M
PRD
OR
W4/W5-like
W1/W2-like
W3-like
YRD
0.005
NS
